# Correction to “Ligand Chromophore Modification Approach for Predictive Incremental Tuning of Metal–Organic Framework Color”

**DOI:** 10.1021/acs.chemmater.3c03160

**Published:** 2024-01-19

**Authors:** Zoe M. Soilis, Tae Hoon Choi, Joe Brennan, Renee R. Frontiera, J. Karl Johnson, Nathaniel L. Rosi

On page 7744, the following changes apply:

- The word “equally” should be removed from the following sentence: “The HOMO-1 energy is equally stabilized when the nitro group is substituted at either the fourth or fifth position on the salicylaldehyde ring.”
- In the sentence “The LUMO energy is stabilized by −0.17 eV when the nitro group is in the fifth position”, “–0.17 eV” should be changed to “–0.35 eV”.
- In the sentence “However, when the nitro group is in the fourth position, the LUMO is highly stabilized by −0.77 eV, significantly lowering the HOMO-1–LUMO gap and shifting the absorption spectrum to a lower energy”, “–0.77 eV” should be changed to “–0.76 eV”.
- In the sentence “However, when the hydroxyl group is in the fifth position, HOMO–1 is highly destabilized by +0.45 eV, significantly lowering the HOMO-1–LUMO gap and shifting the absorption spectrum to a lower energy”, “+0.45 eV” should be changed to “+0.47 eV”.

These errors do not affect the calculated values for orbital energies or conclusions in the paper.

## Supporting Information Available

MOF characterization, including TEM images, PXRD, and gas adsorption studies; computational details; and NMR spectra of MOF products (PDF)

