# Supplementary material for: Correction to “Ligand Chromophore Modification Approach for Predictive Incremental Tuning of Metal–Organic Framework Color”
Source: Chem Mater. 2024 Jan 19;36(3):1773. doi: 10.1021/acs.chemmater.3c03160 (PMC10870409; doi:10.1021/acs.chemmater.3c03160)
Supplement: Supplementary file 1 — cm3c03160_si_001.pdf [file cm3c03160_si_001.pdf]

Ligand Chromophore Modification Approach for Predictive Incremental Tuning of Metal-Organic Framework Color

Zoe M. Soilis[a], Tae Hoon Choi[b], Joe Brennan[c], Renee Frontiera[c], J. Karl Johnson[b], Nathaniel L. Rosi[a,b*]

[a] *Department of Chemistry, University of Pittsburgh, Pittsburgh, PA, 15260, United States*

[b] *Department of Chemical & Petroleum Engineering, University of Pittsburgh, Pittsburgh, PA, 15260, United States*

[c] *Department of Chemistry, University of Minnesota, Minneapolis, MN, 55455, United States*

*Email: nrosi@pitt.edu

Table of Contents

1. H_2-$(NH_2)_2$-TPDC NMR 2
2. Characterization of $(NH_2)_2$-UiO-68 4
  2.1 Transmission Electron Microscopy (TEM) 4
  2.2 Powder X-Ray Diffraction (PXRD) 5
  2.3 Solvent Exchange, Sample Activation, and N_2 Sorption Experiments 6
3. Computational Studies 11
4. Characterization of Product MOFs 20
  4.1 Color of Aldehydes and Product MOFs 20
  4.2 PXRD 21
  4.3 Electron Microscopy Analysis 22
  4.4 ^{1}H NMR Analysis & Quantification of Percentage of Amino Groups Modified 25
  4.4 UV-Vis Absorption 48
5. References 49

1. H_2-$(NH_2)_2$-TPDC NMR

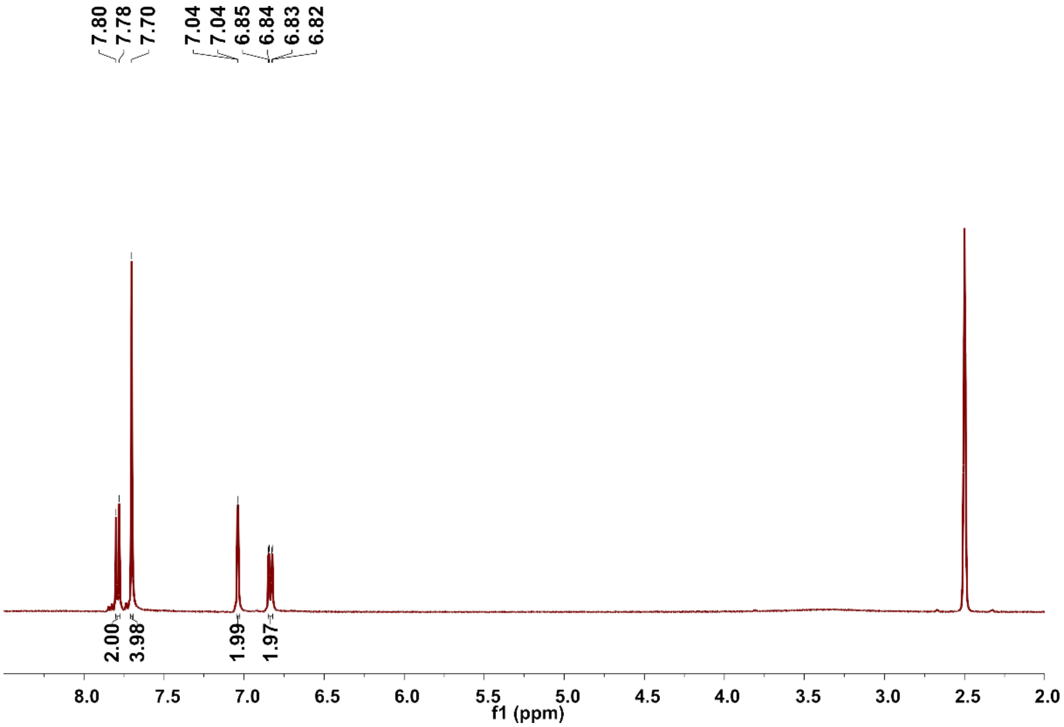

Figure S1A. ^{1}H NMR of 3,3”-diamino-1,1’:4,1”-terphenyl-4,4”-dicarboxylic acid.

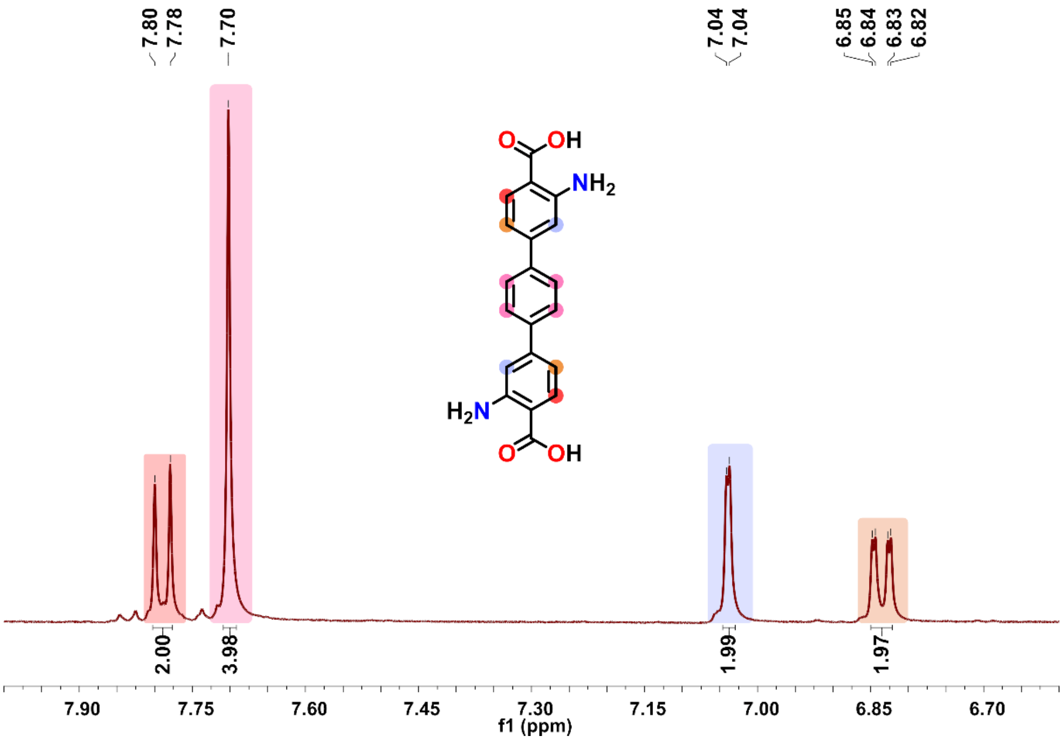

Figure S1B. ^{1}H NMR aromatic region of 3,3”-diamino-1,1’:4,1”-terphenyl-4,4”-dicarboxylic acid.

2. Characterization of $(NH_2)_2$-UiO-68

2.1 Transmission Electron Microscopy (TEM)

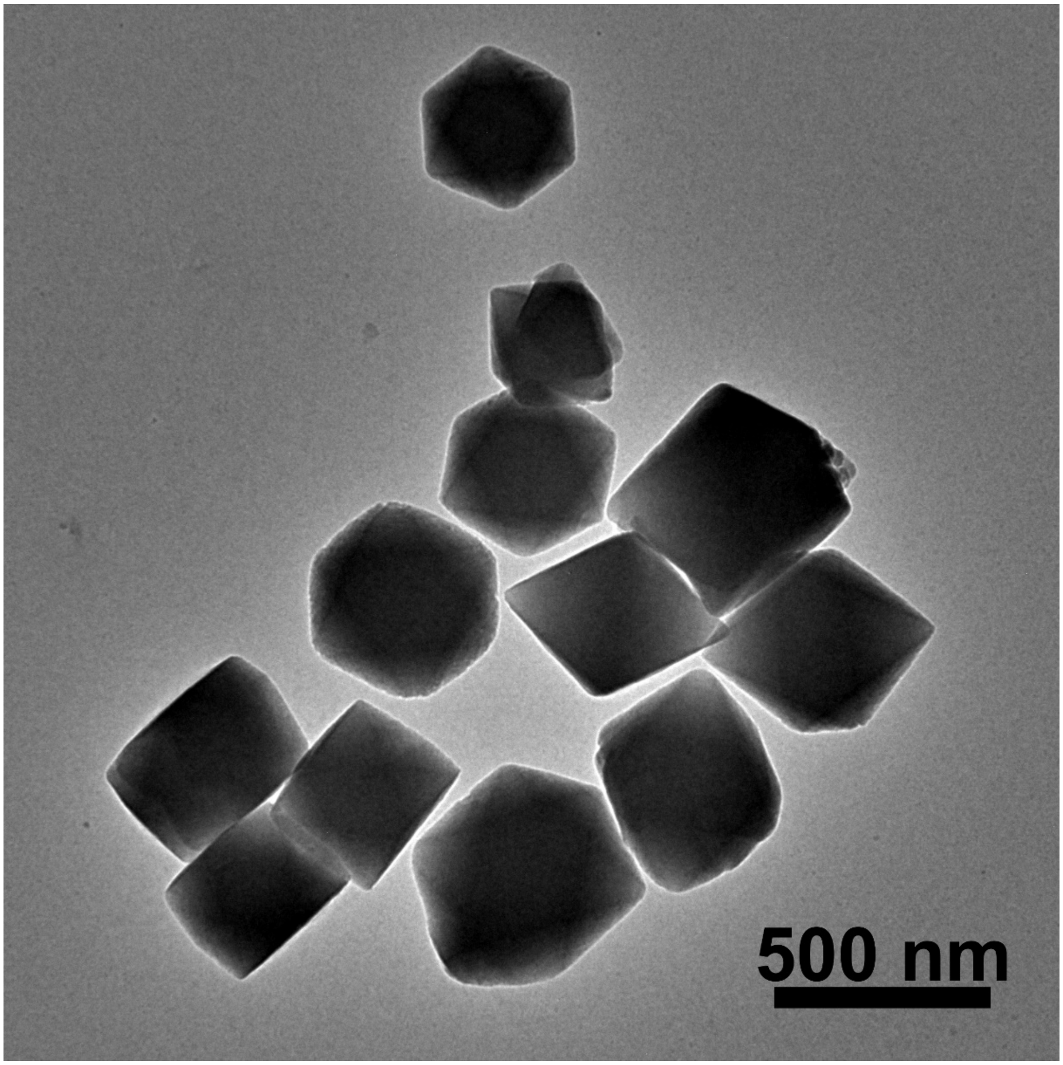

Figure S2. TEM image of $(NH_2)_2$-UiO-68.

2.2 Powder X-Ray Diffraction (PXRD)

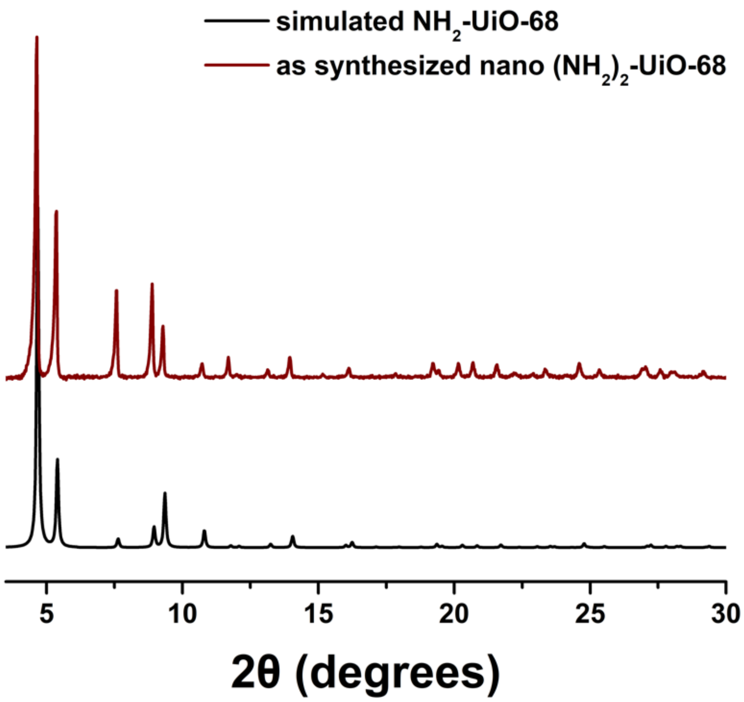

Figure S3. Experimental PXRD pattern of as synthesized $(NH_2)_2$-UiO-68 (red), and simulated PXRD pattern of isostructural NH_2-UiO-68[1] (black) based on its crystal structure.

2.3 Solvent Exchange, Sample Activation, and N_2 Sorption Experiments

Three batches of as-synthesized $(NH_2)_2$-UiO-68 were prepared, thoroughly washed with dry DMF, and then combined in a 50 mL Falcon tube. Solvent exchange was performed with dry chloroform and then pentane using a slightly modified procedure from a literature report.[2] First, excess DMF was removed by centrifuging the sample at 10 000 rpm for 5 mins and decanting the supernatant. Then, the vial was replenished with fresh exchange solvent and placed in a 50°C oven. This solvent exchange procedure was performed every 20 min, with dry chloroform (9 mL each time) for the first 5 cycles and then pentane (18 mL each time) for another 5 cycles. After the final solvent exchange cycle with pentane, the sample centrifuged (10 000 rpm, 5 min), the solvent was decanted, and the sample was gently dried under nitrogen flow.

Even after the solvent exchange procedure, some residual DMF remains in the pore space of the MOF as observed in the ^1H NMR of the digested MOF (Figure S4). Based on analysis of the ^1H NMR data, there are approximately 0.45 molecules of DMF per 1 molecule of linker remaining in the pore space.

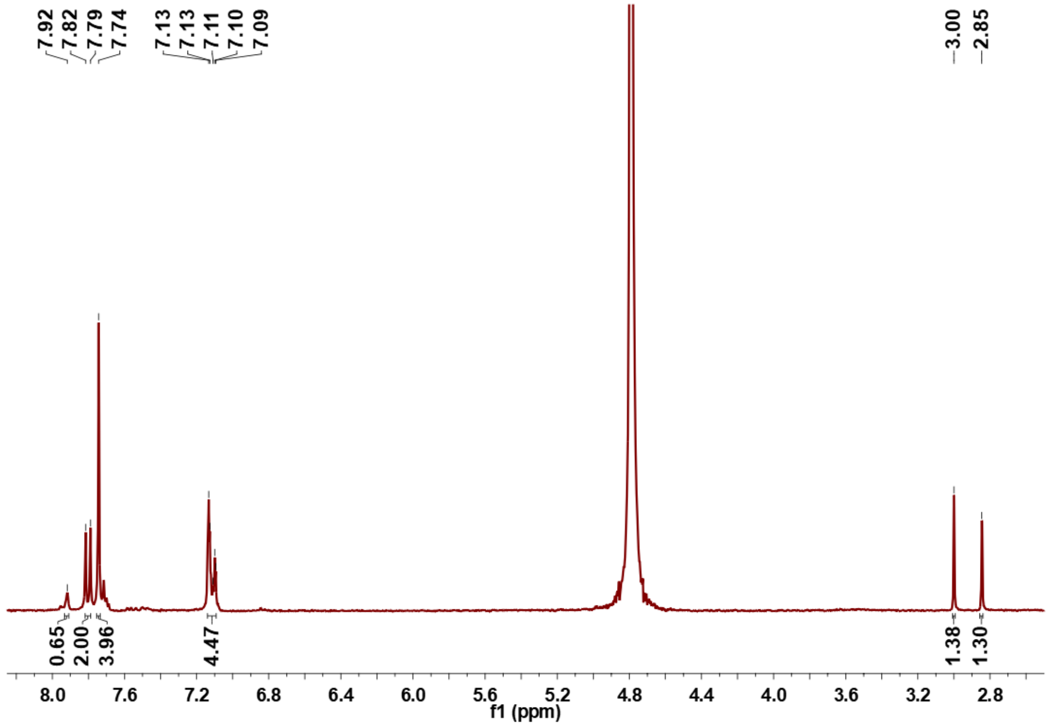

Figure S4. Solution ^1H NMR spectrum of digested solvent-exchanged $(NH_2)_2$-UiO-68. DMF peaks appear at 3.00 (3H), 2.85 (3H), and 7.92 (1H) ppm; DMF:linker ratio = 0.45:1.

After completing the solvent exchange procedure, a MOF sample was evacuated on a Micromeritics Smart VacPrep at room temperature for 21 hours to obtain 15.4 mg of activated sample (note: in our hands, activation under elevated temperatures 100 or 120°C resulted in significant loss of crystallinity, which is why we used room temperature activation). The N_2 sorption isotherm was collected at 77K on a Micromeritics 3Flex instrument.

We used the RASPA software package[3] to simulate the N_2 adsorption isotherm at 77 K within the grand canonical Monte Carlo (GCMC) ensemble. We simulated adsorption at 38 different pressures ranging from 10^{-7} to 101430 Pa. We used the TraPPE N_2 potential[4] and our calculated equilibrium structure of $(NH_2)_2$-UiO-68. The charges on each of the framework atoms were computed from the DDEC6 formalism using the Chargemol program.[5, 6] We estimated the surface area by applying the Brunauer-Emmett-Teller (BET) method[7] to three different pressure ranges,[8, 9] as shown in Table S1. The surface area ranges from 3649 to 4040 m^2/g. These values are much larger than the experimentally calculated BET surface area of 2800 m^2/g (Figure S5). We note that $(NH_2)_2$-UiO-68 loses some crystallinity during the solvent exchange procedure (Figure S6), which could account for the lower-than-expected BET surface area. We emphasize that, in our hands, this MOF seems to be sensitive to solvent exchange and activation conditions; not all sample activation attempts yielded microporous material.

Table S1. Computed BET Surface Areas from N_2 isotherm data at 77 K using GCMC simulations.

Pressure Range (P/P_0)	Surface Area (m^2/g)
0.05 – 0.25	4040
0.05 – 0.30	3927
0.1 – 0.35	3649

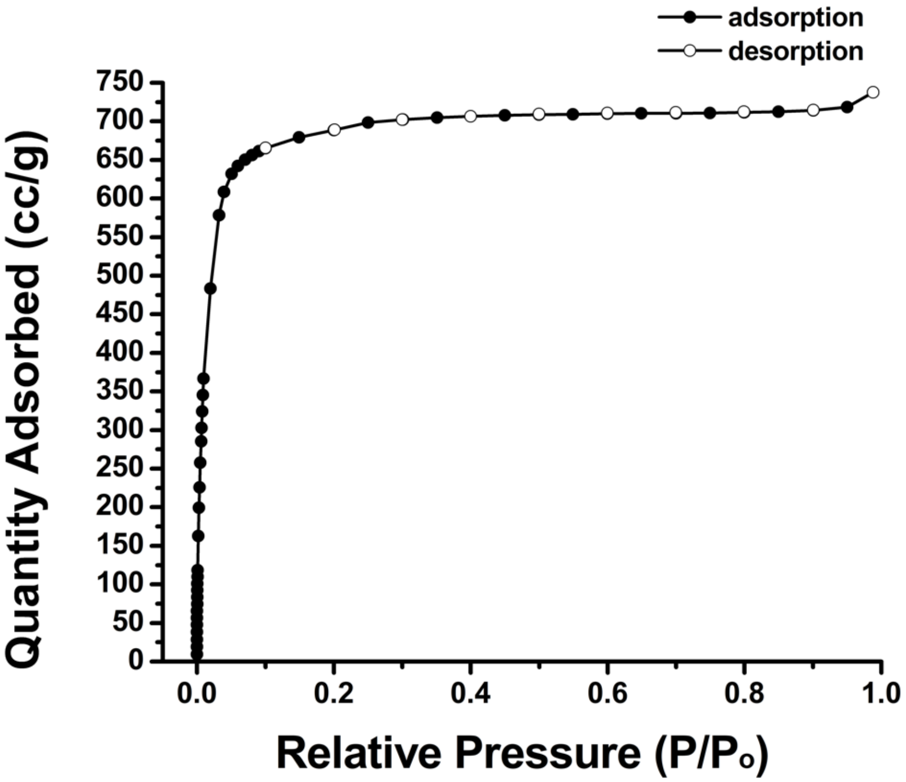

Figure S5. N_2 sorption isotherm at 77 K of $(NH_2)_2$-UiO-68. The calculated BET surface area is 2800 m^2/g.

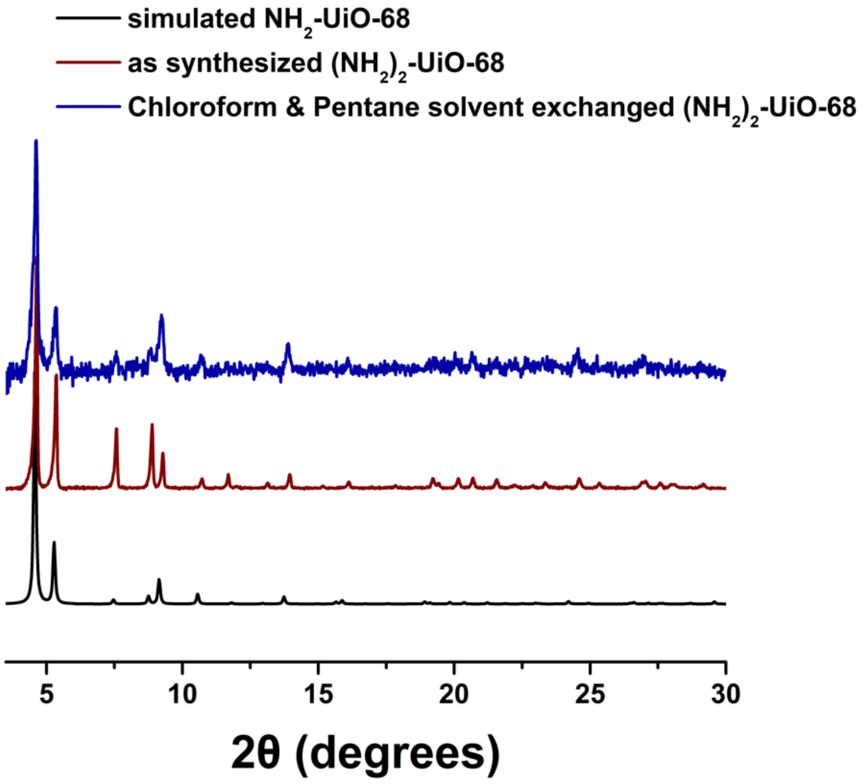

Figure S6. Simulated PXRD pattern of NH_2-UiO-68 (black), as synthesized $(NH_2)_2$-UiO-68 (red) and $(NH_2)_2$-UiO-68 after solvent exchange with dry chloroform and pentane (blue).

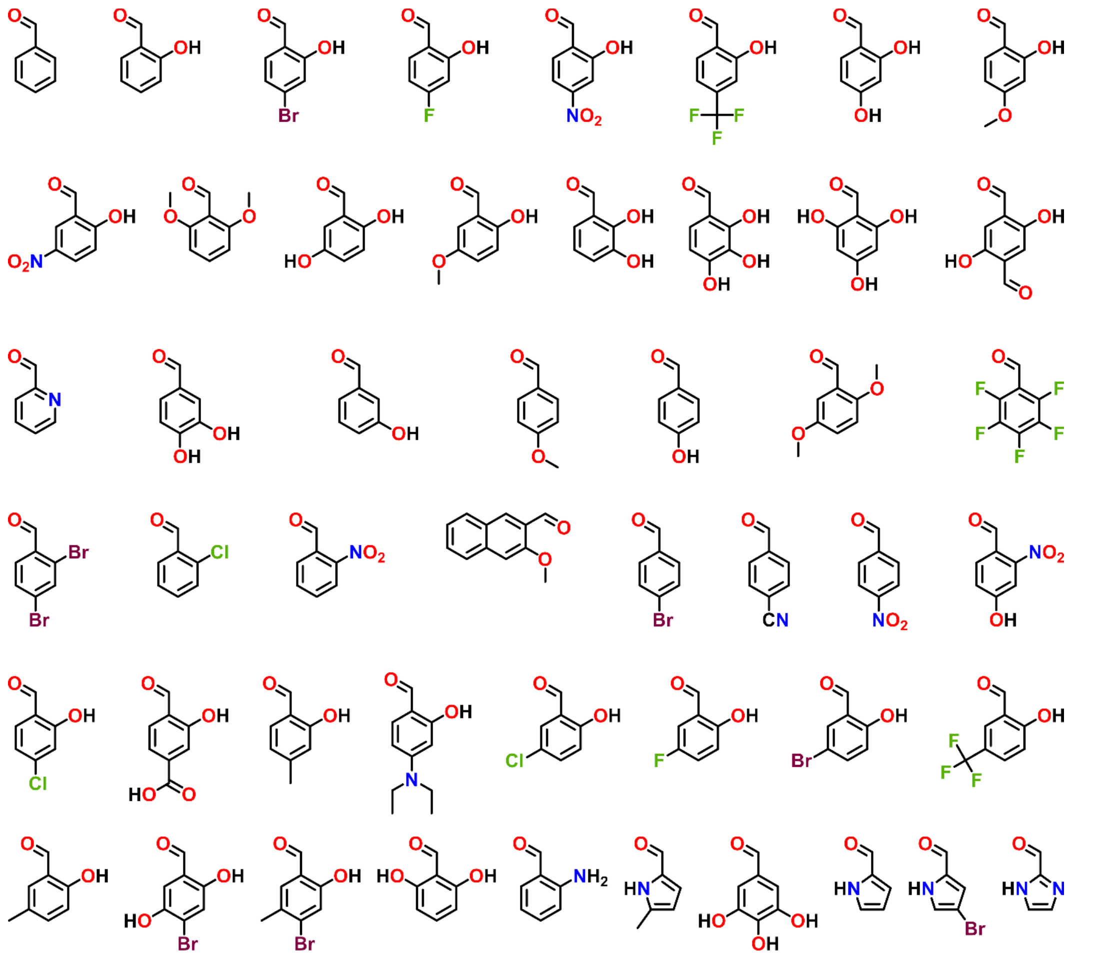

Figure S7. Commercially available aldehydes considered for use as linker chromophore modifiers.

3. Computational Studies

We have computed the UV-Vis spectra for the H_2-$(NH_2)_2$-TPDC linker and its modified analogues by applying time-dependent density functional theory (TD-DFT) to compute excited state properties. As benchmark calculations, we examined the vertical singlet electronic excitation energies using non-hybrid, hybrid, and range-separated hybrid DFT. We also investigated the HOMO-LUMO gap, which provides only the fundamental gap, the energy difference between the ionization potential and electron affinity of the molecules.[10] The HOMO-LUMO energy gap is usually higher than the electronic excitation energy because the fundamental gap does not consider Coulomb and nonlocal exchange potentials between the HOMO and LUMO.[11] Furthermore, it has been shown that the orbital energy gap highly depends on the specifics of the computational methodology. Although the fundamental gap is higher than an optical gap, it can be useful for the prediction of the relative electronic transition energies for the derivatives of the molecules.

Figure S8 shows UV-Vis spectra of the modified ligand, **C**, calculated with different DFT functionals: generalized gradient approximation Perdew-Burke-Ernzerhof+D3 (PBE+D3), hybrid B3LYP+D3, and range-separated hybrid ωB97XD. Both the line peaks (black lines) and the Gaussian band shape (red solid line) are shown in Figure S8. The equation for the Gaussian band shape is:

$$\varepsilon(\tilde{v}) = \sum_{i=1}^{n} \varepsilon_i(\tilde{v}) = \sum_{i=1}^{n} \left(1.3062974 \times 10^8 \cdot \frac{f_i}{\sigma} \exp\left[-\left(\frac{\tilde{v} - \tilde{v}_i}{\sigma}\right)^2\right]\right),$$

where the i subscript refers to the electronic excitation of interest, $\tilde{v}_i$ and f_i are the excitation energies and the oscillator strengths, respectively. A standard deviation (σ) of 0.2 eV is used in this plot. All the calculations were performed using the Gaussian 16 program package.[12]

For the non-hybrid functional, PBE+D3, two peaks around 480 nm appear with very small oscillator strengths, and other peaks are distributed between 300 and 450 nm. For the hybrid functional, B3LYP, most of the line peaks are blue-shifted relative to the PBE+D3 results. In case of the range-separated DFT (ωB97XD), the peaks are more blue-shifted, and the Gaussian band shape shows three separated main peaks, which is similar to the experimental absorption spectra in Figure 4C. Thus, by applying different functionals in the TD-DFT calculations, the band shapes are changed to be more clearly separated and blue-shifted in the order of PBE+D3, B3LYP+D3, and ωB97XD. Based on the benchmark calculation, we used the ωB97XD method for the HOMO-LUMO orbital analysis and the electronic excitation energies.

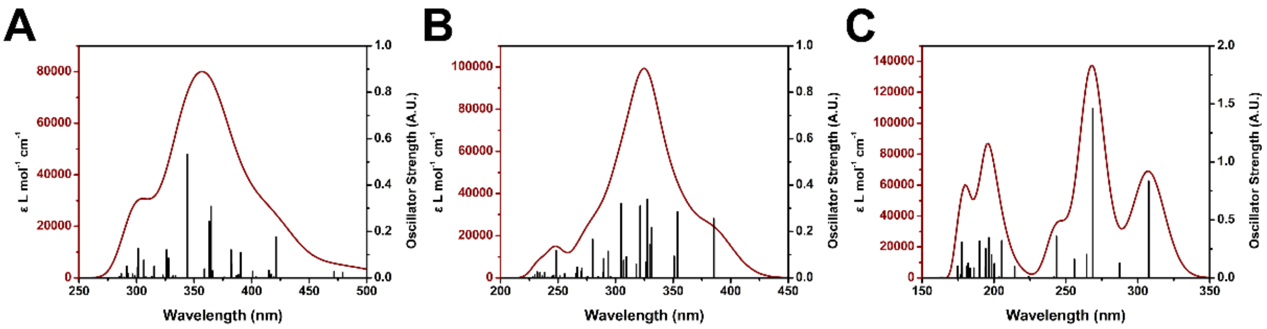

Figure S8. UV-Vis spectra of **C** calculated with a non-hybrid functional, PBE+D3, (A); a hybrid functional, B3LP+D3, (B); and a range-separated hybrid functional, ωB97XD, (C) level of theory with the 6-31G(d) basis set.

To examine the impact of periodic boundary conditions on the calculated UV-Vis spectra, we optimized periodic **1-C** at 25% modification using the Perdew-Burke-Ernzerhof exchange-correlation functional revised for solids (PBEsol) density functional with Goedecker-Teter-Hutter pseudopotentials and the DZVP-MOLOPT basis set, including Grimme's D3 dispersion correction using the CP2K software package.[13-16] The atom positions of the $(NH_2)_2$-UiO-68 primitive cell, using lattice parameters a = b = c = 23.19722 Å, α=β=γ= 60°, were relaxed using DFT in CP2K. The computational UV-Vis spectra of the periodic **1-C** were calculated using the time-dependent density functional perturbation theory (TD-DFPT) technique combined with the Gaussian and Augmented-Plane-Wave (GAPW) procedure.[17] Figure S9 shows the computed UV-Vis spectra of **1-C** at the optimized structure of the primitive cell, in which 25% of the amino groups are modified. There are many peaks between 330 nm and 380 nm and the peaks gradually decay until 600 nm. These peaks are red-shifted compared to the calculated UV-Vis spectra of the ligand in the gas phase (Figure S8) and compared with the experimental spectra for **1-C** (Figure 4). This red shift is likely due to the use of the non-hybrid PBEsol functional. Note that it is not feasible to use a hybrid or range-separated functional for the periodic calculation because of the excessive computational time required. The GAPW procedure provides a useful DFT technique for the calculation of electronic excitation spectra of large systems in the condensed phase. However, it does not have the capability for transition orbital analysis, which allows precise identification and visualization of the electronic excitations.[18] Thus, we have used the DFT and TD-DFT calculations of the molecular ligands for the electronic transition and orbital analysis.

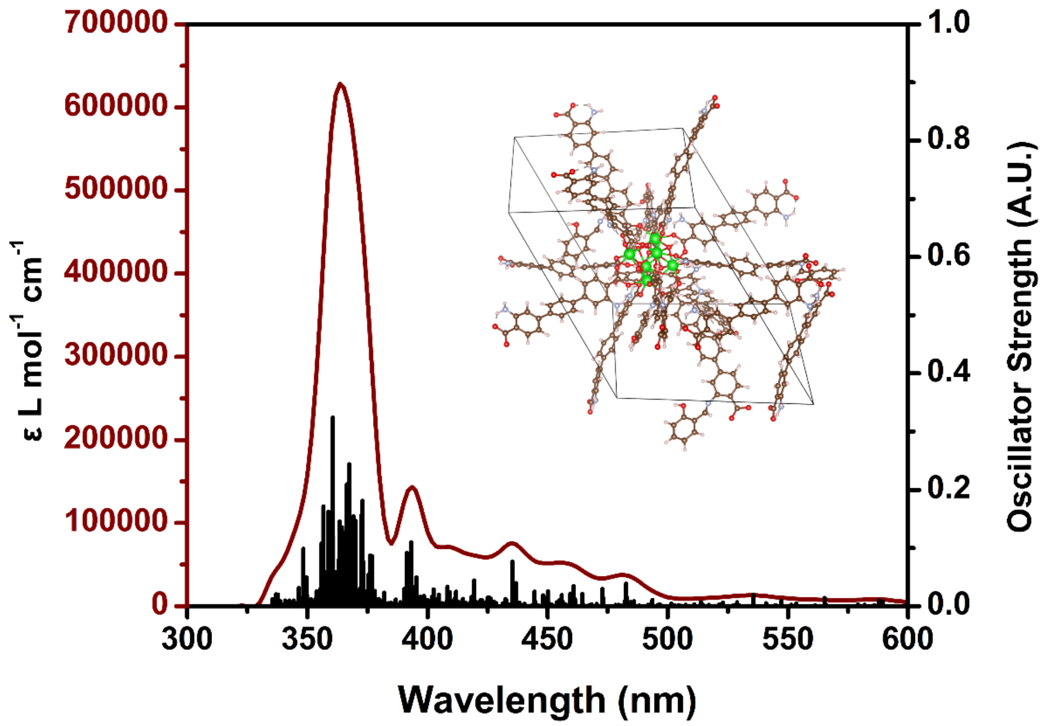

Figure S9. The optimized structure of the **1-C** modified at 25% (inset image) and UV-Vis spectrum of the optimized structure calculated the TD-DFPT method.

We have also calculated the computational UV-Vis spectra of **C**, with three different basis sets, 6-31G(d) (560), aug-cc-pVDZ (962), and cc-pVTZ (1300), where the number of basis functions is given in parentheses. Our results are plotted in Figure S10. For the larger basis sets, the peaks are slightly red-shifted, but the shapes of all the peaks are very similar, indicating the basis set effect is minor. We have selected the 6-31G(d) basis set for the HOMO-LUMO analysis and TD-DFT calculations on the basis of computational efficiency.

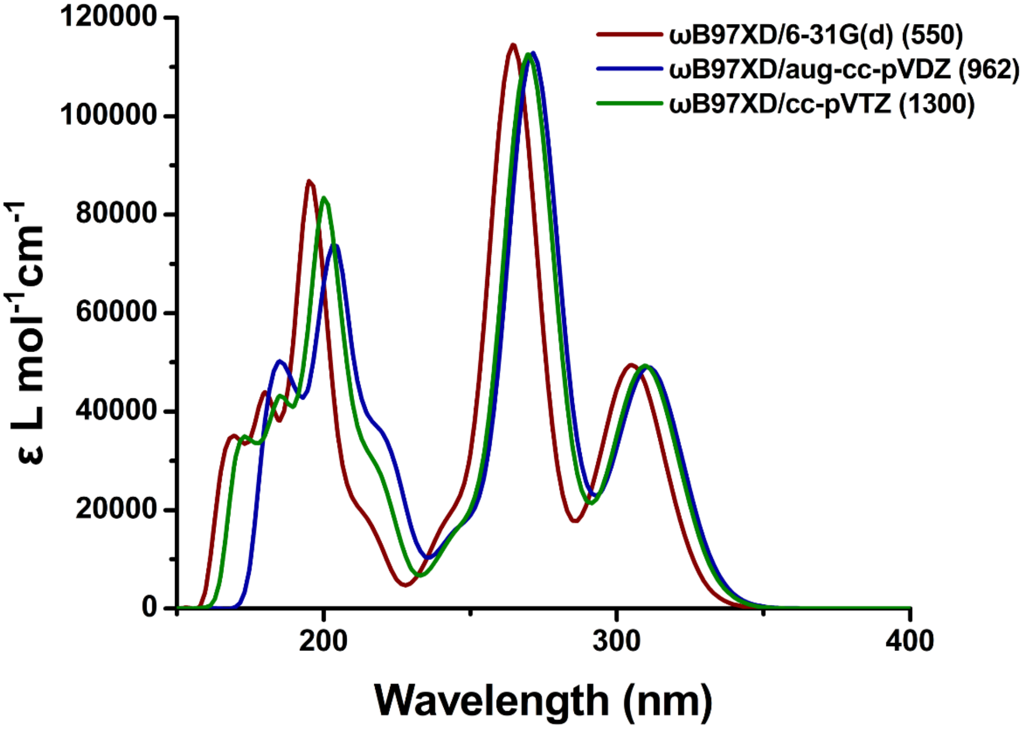

Figure S10. UV-Vis spectra of **C** calculated with ωB97XD using 3 different basis sets: 6-31G(d), aug-cc-pVDZ, and cc-pVTZ.

From the experimental analysis, ~33% of the amino groups were modified. One might expect that 100% modified, 50% modified, and unmodified linkers co-exist in the crystal. Thus, it is instructive to analyze the UV-Vis spectra for each case. Figure S11 shows the UV-Vis spectra for the three initially selected ligands, **A**, **C**, and **F** (Scheme 1). In the UV-Vis spectra of 100% modified ligands, the peaks between 280 nm and 360 nm are clearly distinguished for each linker (Figure S11A). For the 50% modified ligands (Figure S11B), the peak intensities between 280 nm and 360 nm are decreased because of the 50% modification. The entire shapes of the UV-Vis spectra of **A** and **C** are like those of the 100% modified ligands. Interestingly, for **F**, the peak at 345 nm becomes bimodal when it is 50% modified. These features can be explained by analyzing the HOMO-LUMO transitions.

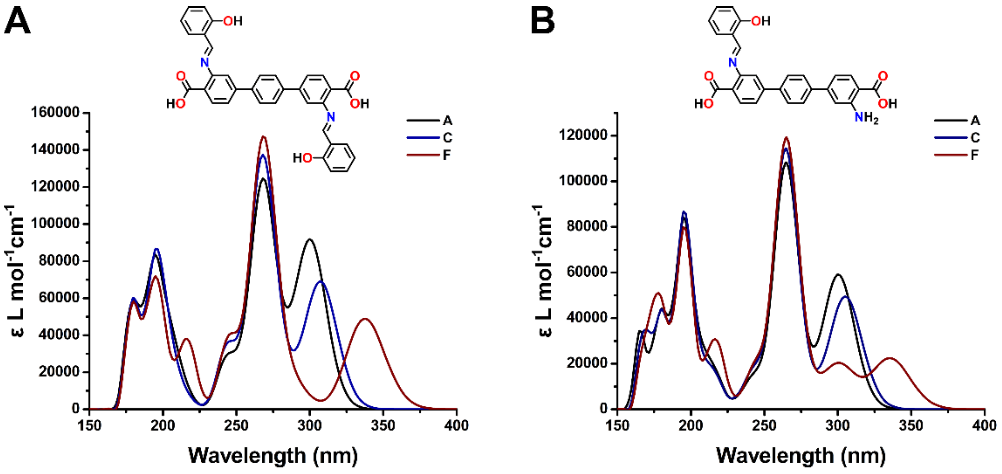

Figure S11. UV-Vis spectra of **A**, **C**, and **F** at 100% (A) and 50% (B) modification.

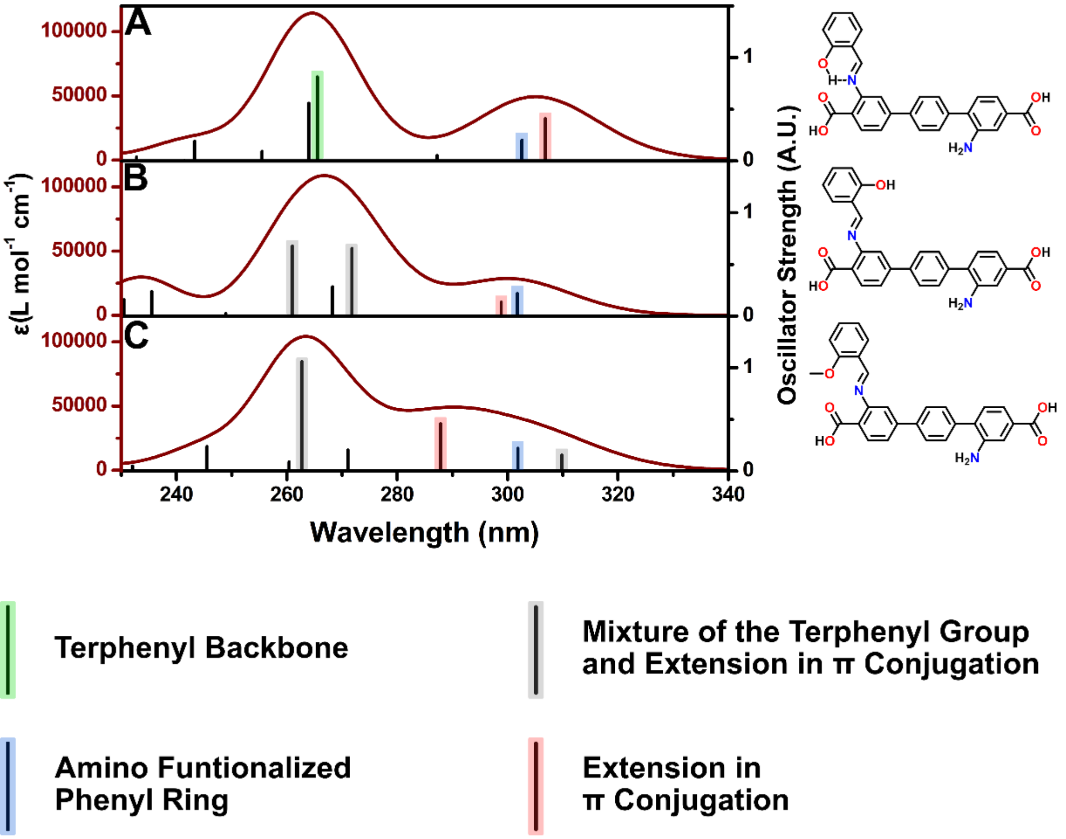

Figure S12. Calculated UV-Vis absorption spectra to determine the influence of ortho hydroxyl group hydrogen bond. **I** (A, top) has an intramolecular hydrogen bond, **II** (B, middle) does not have a hydrogen bond because of rotation of the phenyl ring, and **III** (C, bottom) has a 3-methoxybenzaldehyde group instead of a phenyl group. Transitions associated with specific portions of the modified linker are color-coded.

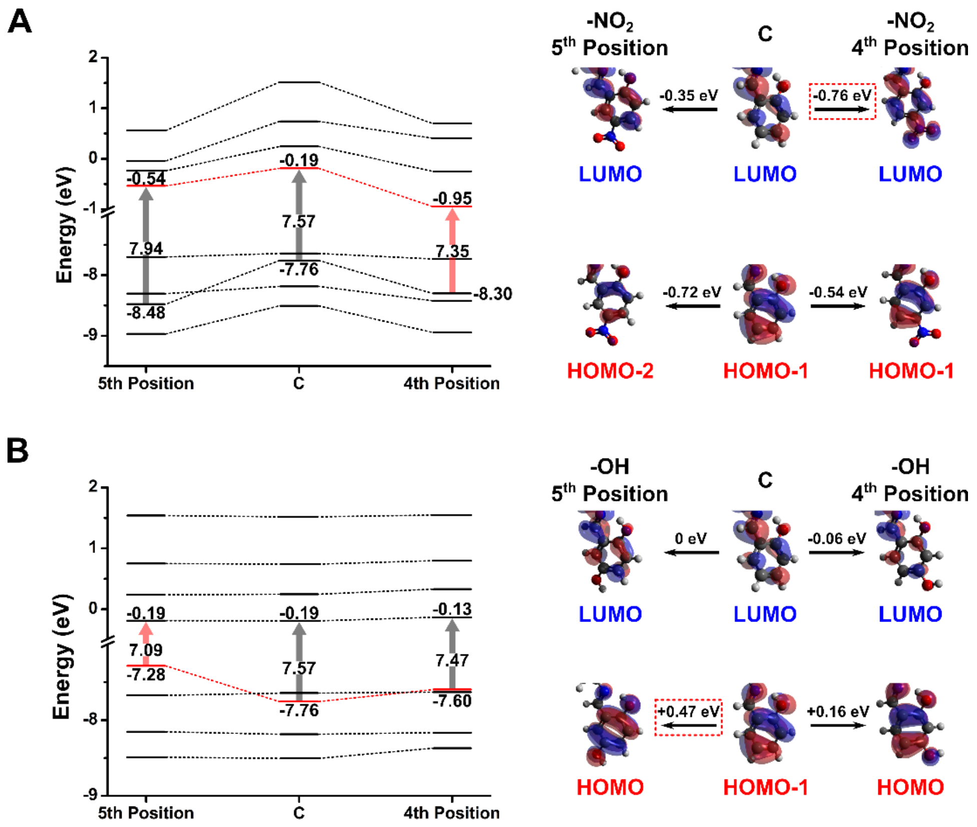

Figure S13. Selected HOMO to LUMO transitions having the largest contributions in the TD-DFT calculations for the electronic transition associated with the extended π-conjugation. (A, left) Calculated molecular orbital energy diagrams of **C** and the ligand modified with a salicylaldehyde modifier having an electron withdrawing nitro group at the fourth and fifth positions. The energies of relevant HOMO and the LUMO and the energy of the transition are listed in the graph. (A, right) Molecular orbital plots and relative differences in molecular orbital energies. The significant decrease in the LUMO energy when the nitro group is in the fourth position is shown in the red box. (B, left) Calculated molecular orbital energy diagrams of **C** and the ligand modified with a salicylaldehyde modifier having an electron donating hydroxyl group at the fourth and fifth positions. The relevant HOMO and LUMO energies are shown, along with the energy of the gap. (B, right) Molecular orbital plots and the relative differences in molecular orbital energies compared to **C**. The significant increase in the HOMO energy when the hydroxyl group is in the fourth position is shown in the red box. Note that the energy ordering of the HOMO is changed when nitro group is substituted at the fourth position or the hydroxyl group is substituted at the fourth and fifth position of the π conjugated ring.

Figure S14 shows the computational UV-Vis spectra with both line peaks and Gaussian band shape for the 50% modified ligands **A**-**G** as well as H_2-$(NH_2)_2$-TPDC. In H_2-$(NH_2)_2$-TPDC, there are only two peaks. The green highlighted peak is the electronic excitation from the HOMO of the terphenyl group, and the blue highlighted peak is from the amino functionalized phenyl ring. In the 50% modified ligands, another lower energy peak appears, highlighted in red, which is from the HOMO associated with the extended π conjugation. All green and blue highlighted peak positions of the target ligands **A**-**G** are located at almost the same wavelength, ~266 nm and ~303 nm, respectively. However, the red highlighted peaks appear at different wavelengths because all **A**-**G** ligands have different functional groups at different positions on the π conjugated ring, as discussed in the text.

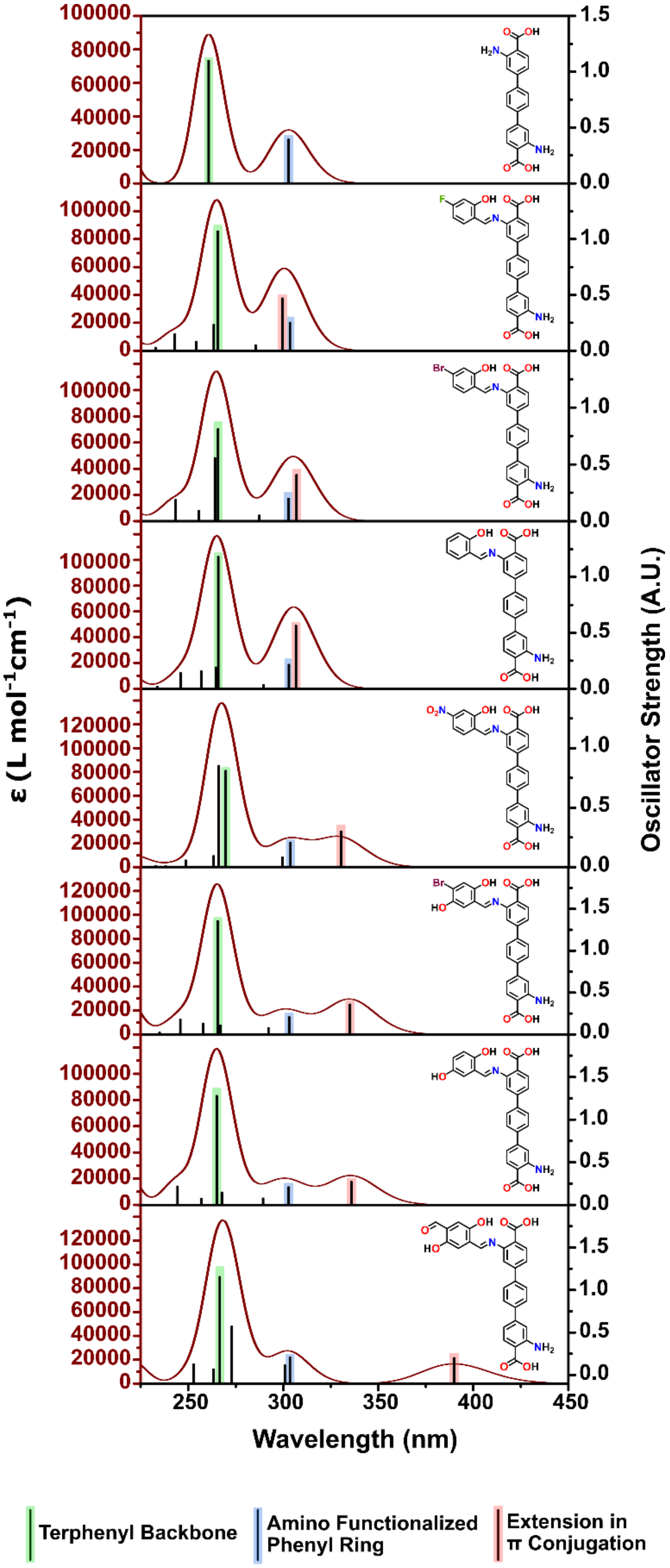

Figure S14. Three different line peak trends in the target modified ligands, **A**-**G**, and H_2-$(NH_2)_2$-TPDC calculated with ωB97XD/6-31G(d).

4. Characterization of Product MOFs

4.1 Color of Aldehydes and Product MOFs

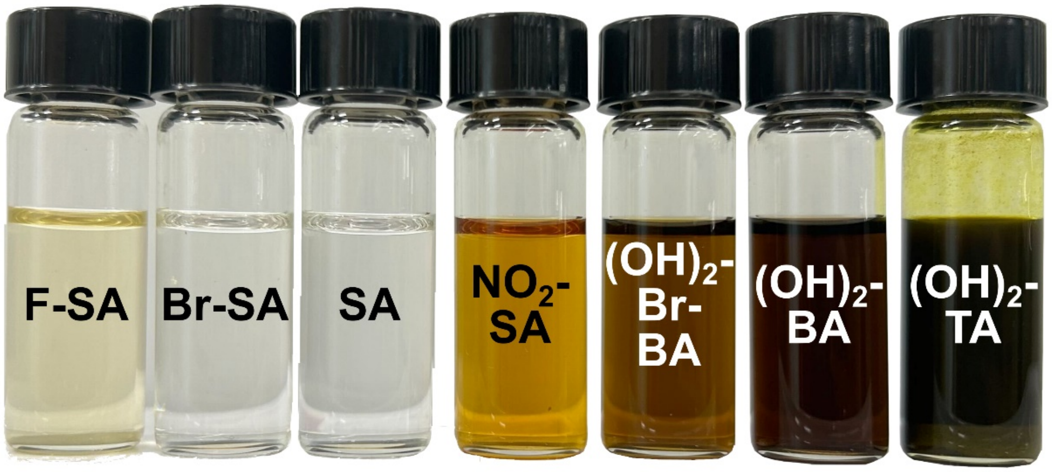

Figure S15. 0.5M DMF solutions of aldehyde modifiers: 4-fluorosalicylaldehyde (**F-SA**) 4-bromosalicyaldehyde (**Br-SA**); salicylaldehyde (**SA**); 4-nitrosalicylaldehyde (**NO2-SA**); 2,5-dihydroxy-4-bromobenzaldehyde [**$(OH)_2$-BA**]; 2,5-dihydroxybenzaldehyde [**$(OH)_2$-Br-BA)**]; and 2,5-dihydroxyterephthaldehyde [**$(OH)_2$-TA**] (from left to right).

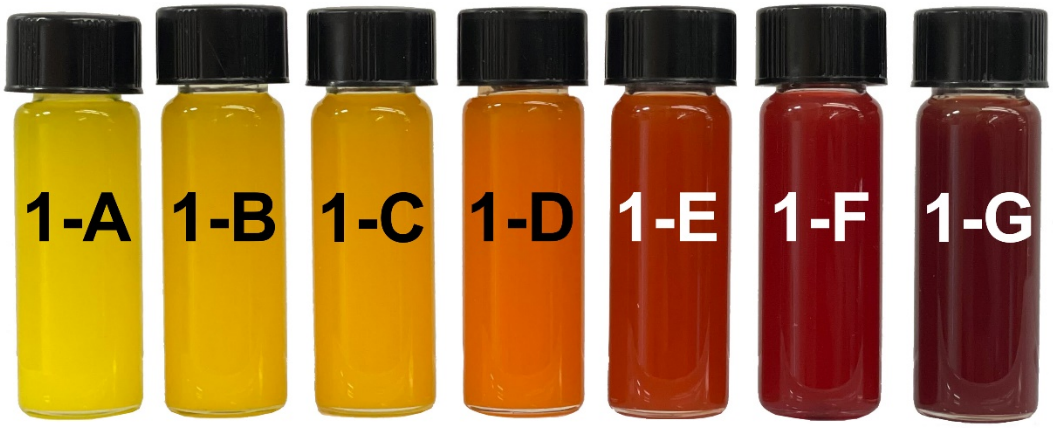

Figure S16. Modified MOFs **1-A** – **1-G** in DMF.

4.2 PXRD

PXRD patterns were collected to confirm that crystallinity was maintained after post-synthetic modification of $(NH_2)_2$-UiO-68.

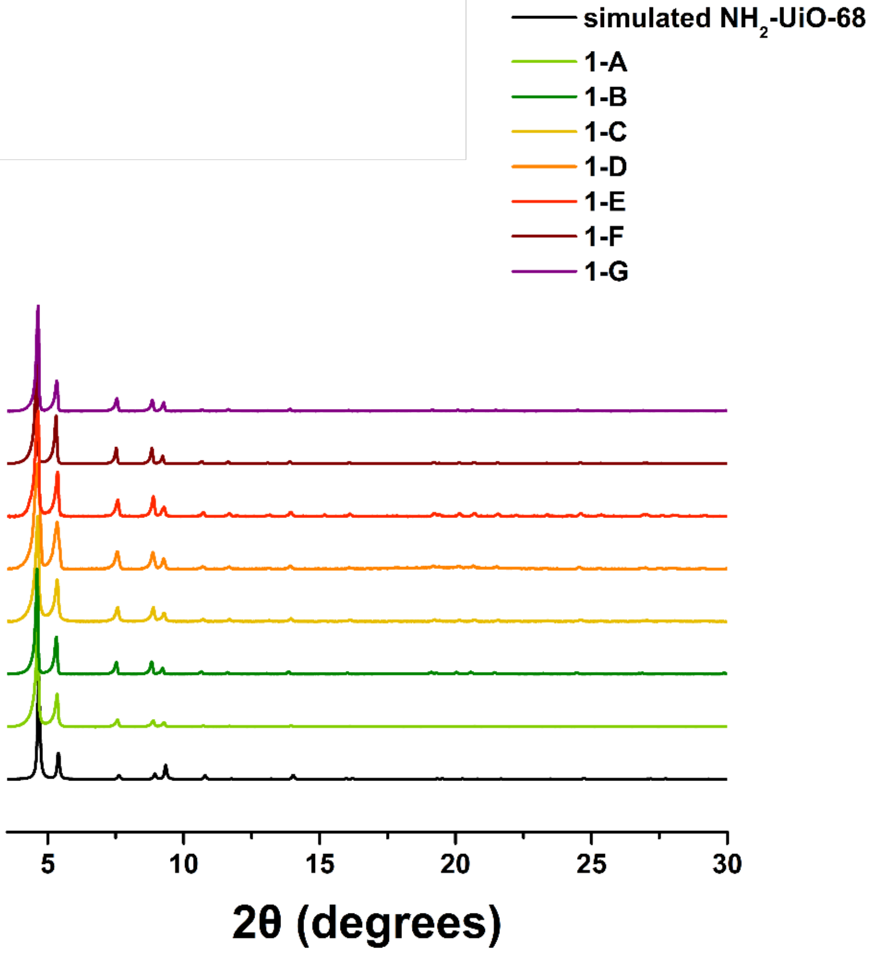

Figure S17. Simulated PXRD pattern of NH_2-UiO-68 (black) and **1-A** – **1-G**. All MOF crystals were washed with dry DMF prior to collecting PXRD patterns.

4.3 Electron Microscopy Analysis

TEM imaging confirms MOF size and morphology is retained after the PSM experiments (Figure S18). STEM-EDS mapping (Figure S19) and line-scan data (Figure S20) of **1-B** indicate that both Zr (from MOF SBU) and Br (from aldehyde modifier) are distributed throughout a single MOF crystal.

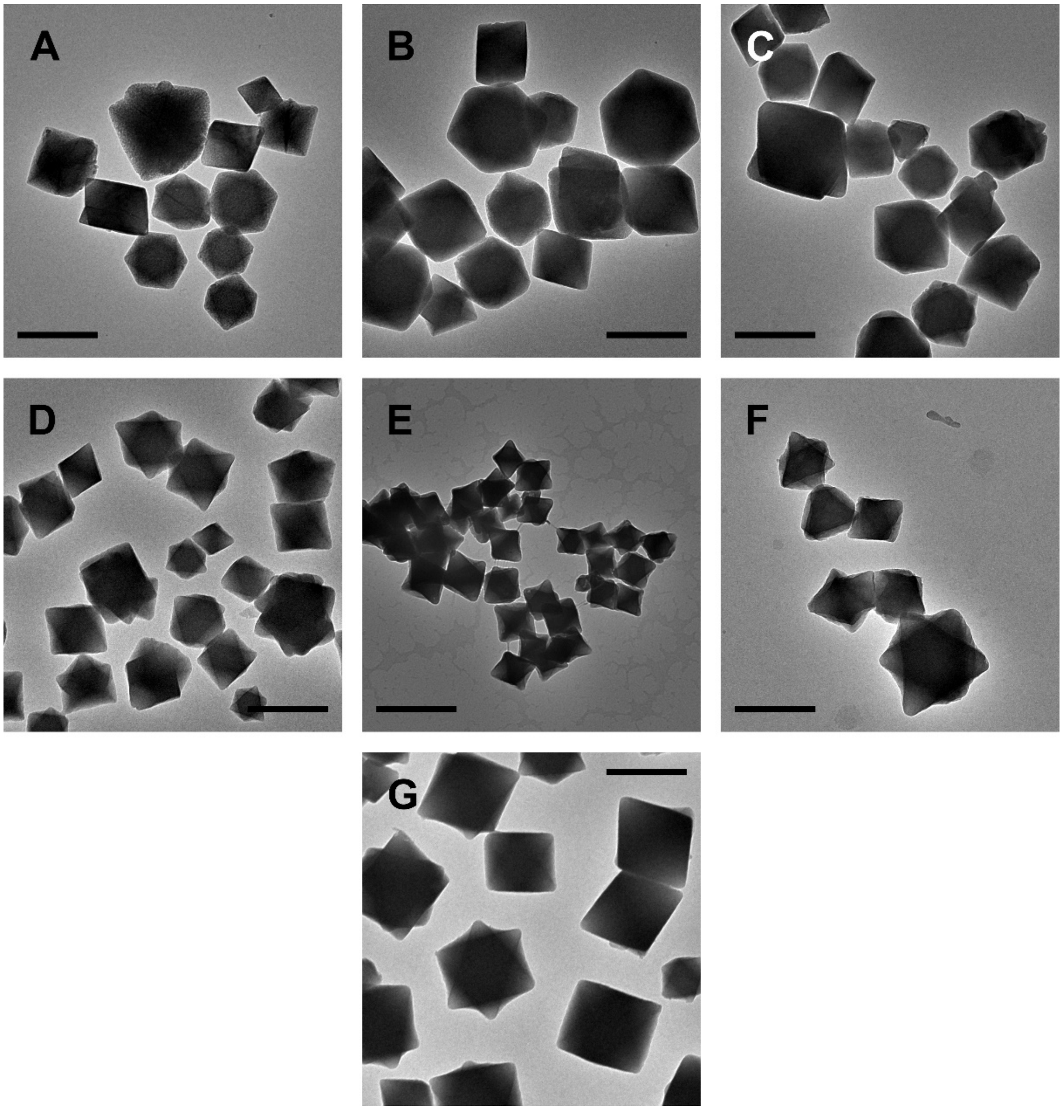

Figure S18. TEM images of modified MOFs **1-A** – **1-G** (A-G, respectively). Scale bars = 500 nm.

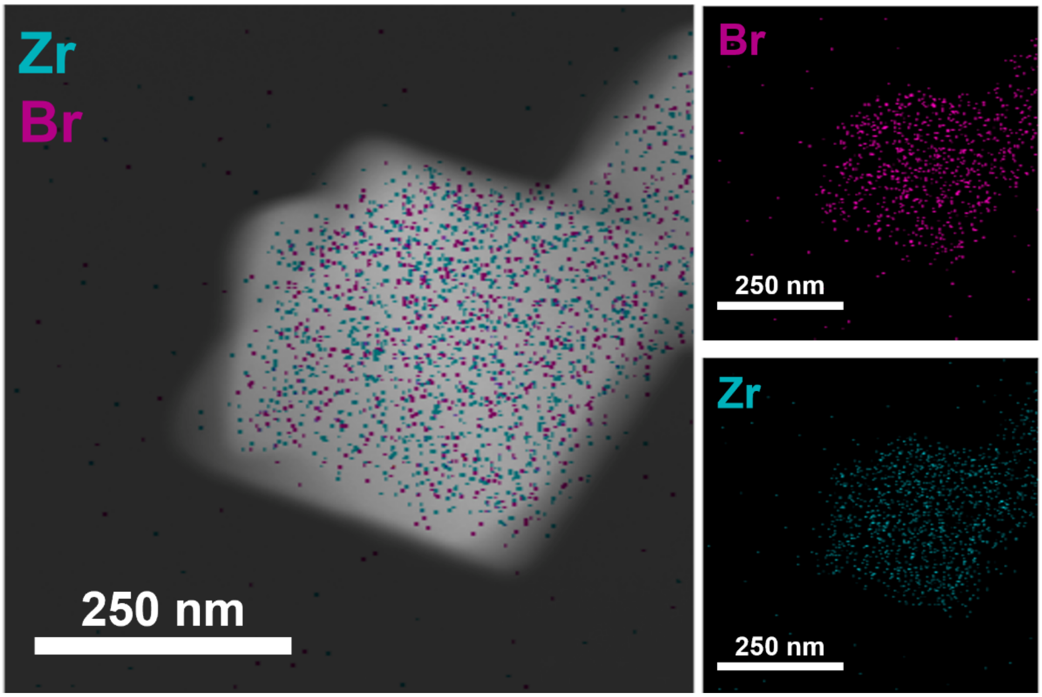

Figure S19. STEM-EDS mapping of **1-B**. EDS Maps were generated using the Zr Kα1 line at 15.7 keV and Br K α1 line at 11.9 keV.

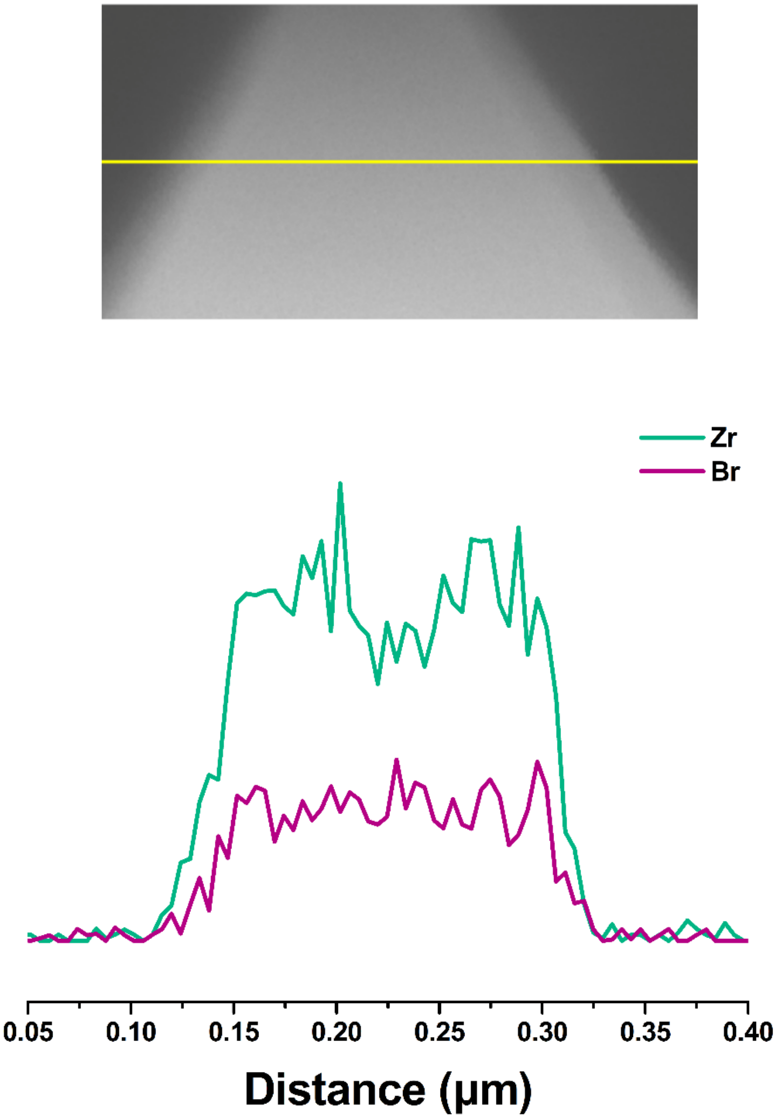

Figure S20. STEM-EDS line-scan data of **1-B**. EDS line-scan data were generated using the Zr Kα1 line at 15.7 keV and Br Kα1 line at 11.9 keV.

4.4 ^{1}H NMR Analysis & Quantification of Percentage of Amino Groups Modified

Initially, we used several strategies to quantify the PSM modification percentage. Due to the instability and possible reversibility of the imine bond with acid and/or excess water, we first tried to reduce the imine before quantification with ^{1}H NMR.[19] Since the MOF linker is functionalized with two amino groups per linker, there are three possible products after the PSM reaction: unfunctionalized, mono-functionalized, and di-functionalized. The three possible products lead to highly complicated ^{1}H NMR spectra, especially in the aromatic region. Therefore, we decided to take advantage of the reversible imine bond to purposefully induce the reverse reaction with the NMR solvent (K_3PO_4 in D_2O) and quantify the MOF linker and aldehyde modifier separately.

After post-synthetic modification and repeated washing with dry DMF (see synthesis in Experimental section of manuscript), the modified MOFs were suspended in 1 mL dry DMF. To prepare each MOF sample for ^{1}H NMR analysis, 0.5 mL of the modified MOF suspension was centrifuged, excess solvent was decanted, and the remaining MOF was dried under vacuum on a Schlenk line. The dried MOFs were digested in 0.5 mL of a potassium phosphate tribasic solution in D_2O (20 mg/mL) and sonicated until all MOF solid was dissolved.

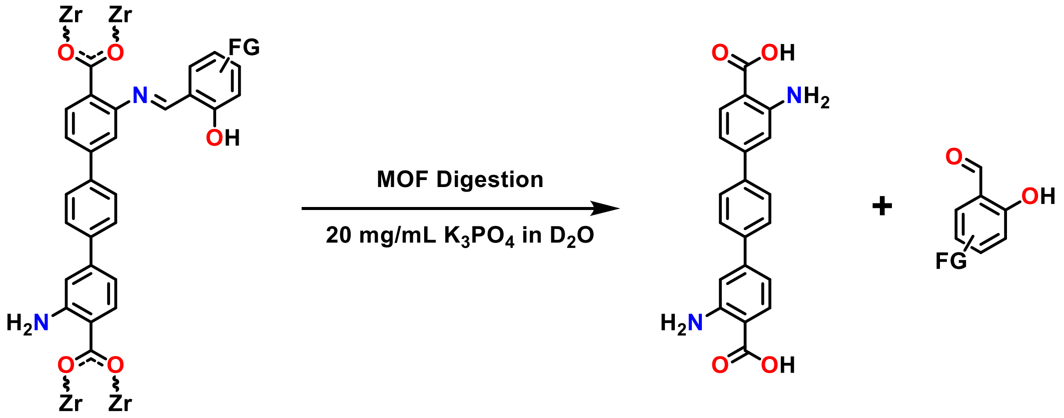

Scheme S1. Reversal of post synthetic modification under basic MOF digestion conditions for ^{1}H NMR.

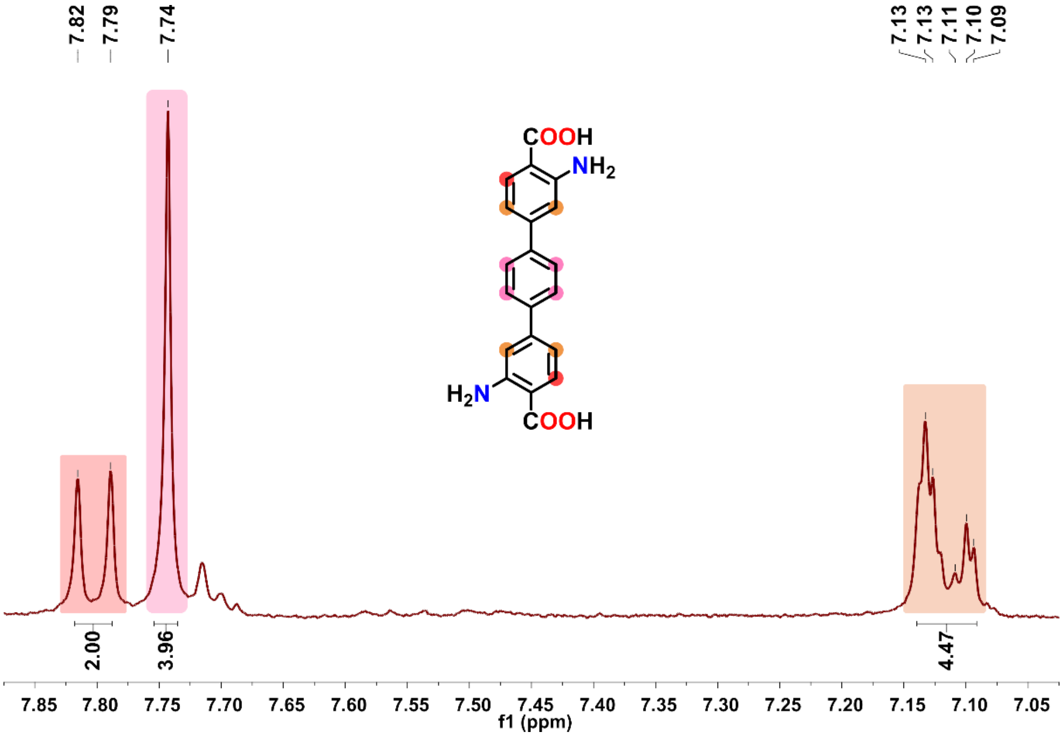

Figure S21. ^{1}H NMR of **1** in K_3PO_4/D_2O (aromatic region).

Table S2. ^{1}H NMR Peaks of Digested **1** for Quantification

Linker Peaks	Position (ppm)	Integration	Protons Per Molecule	Normalization Value
Red	7.81	2.00	2	1.00
Orange	7.11	4.47	4	*
Pink	7.74	3.96	4	0.99
			**Average**	**1.00**

*These peaks are overlapping with other peaks and were not used for quantification

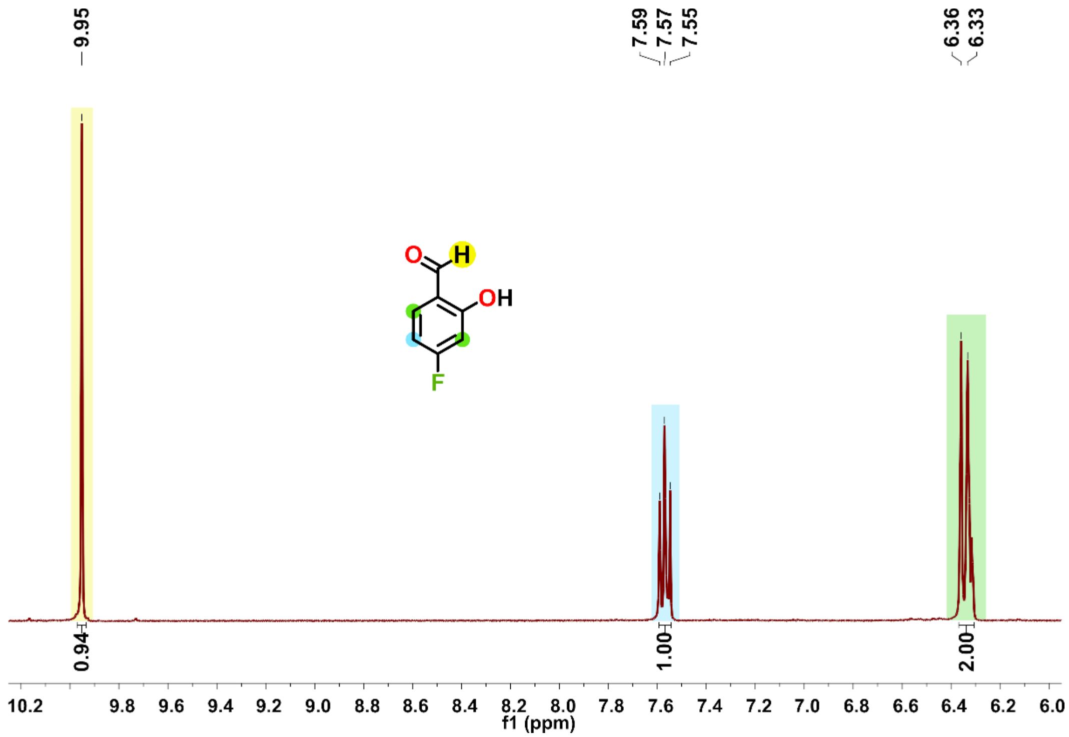

Figure S22. ^{1}H NMR (aromatic and aldehydic proton region) of 4-fluorosalicylaldehyde in K_3PO_4/D_2O.

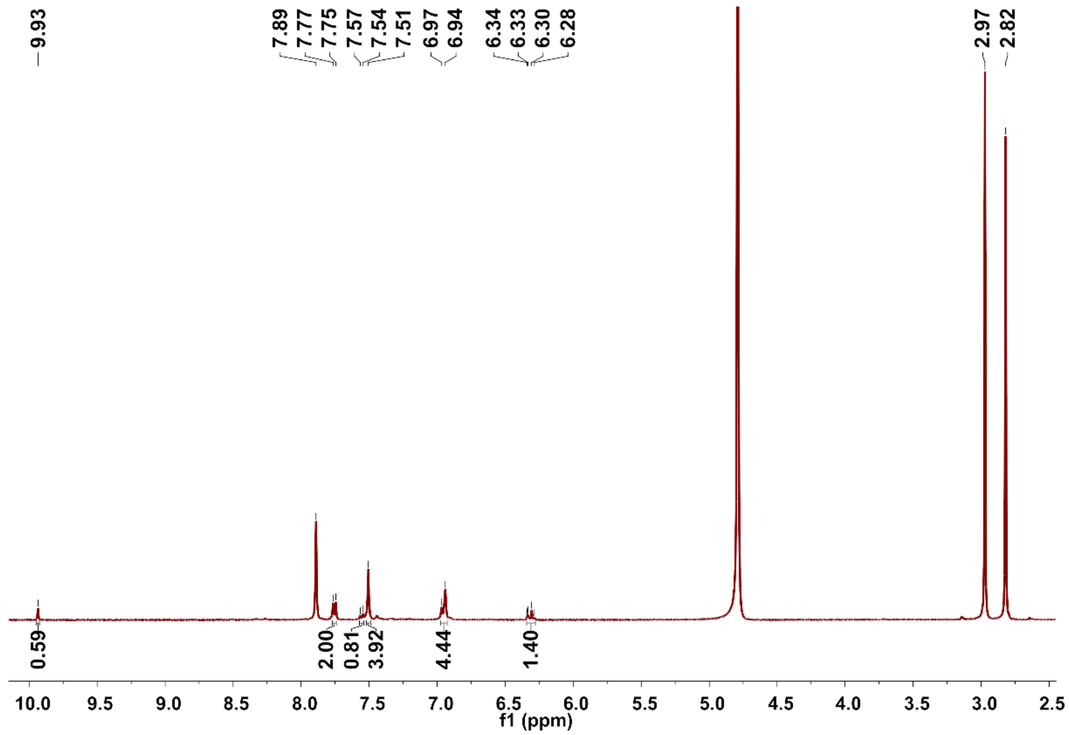

Figure S23. ^{1}H NMR of **1-A** in K_3PO_4/D_2O (peaks at δ = 2.82, 2.97, and 7.89 correspond to the protons in DMF).

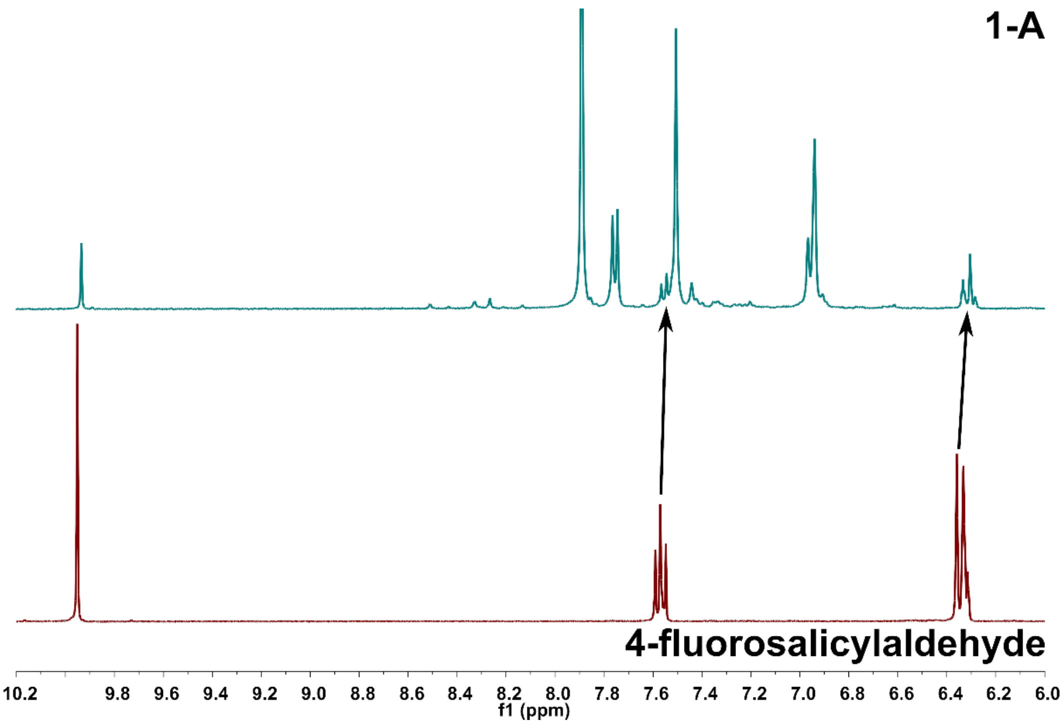

Figure S24. Overlay of ^{1}H NMR (aromatic and aldehydic proton region) of 4-fluorosalicylaldehyde (bottom) and **1-A** (top).

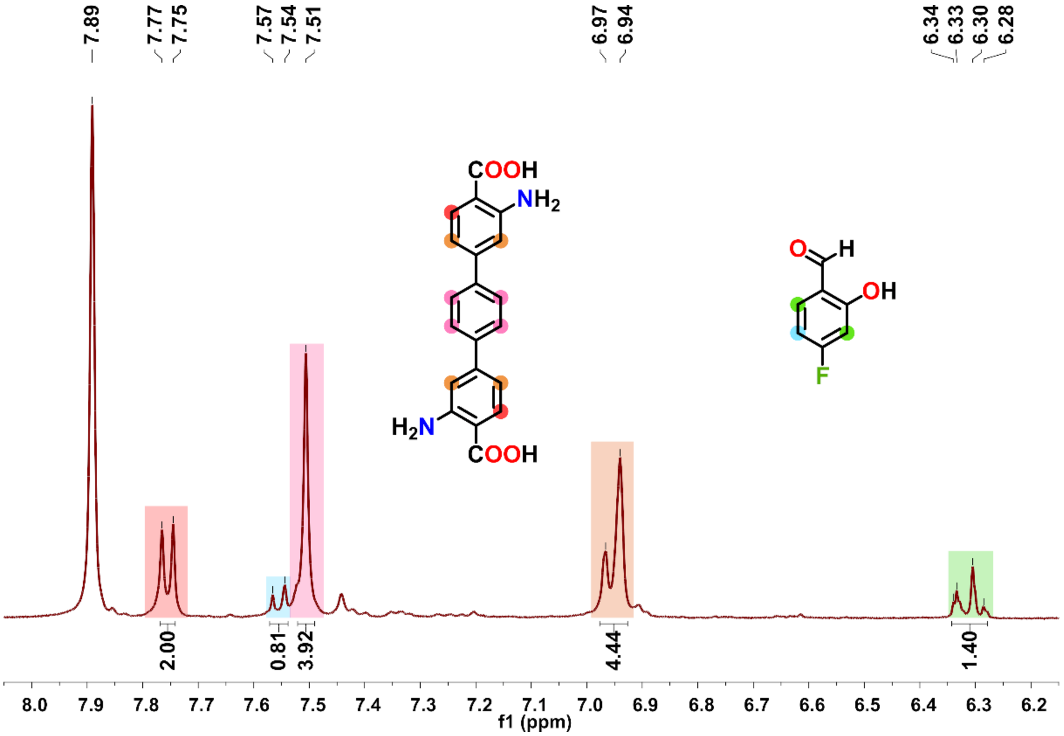

Figure S25. ^{1}H NMR of **1-A** in K_3PO_4/D_2O (aromatic region; peak at $\delta = 7.89$ corresponds to the aldehyde proton in DMF).

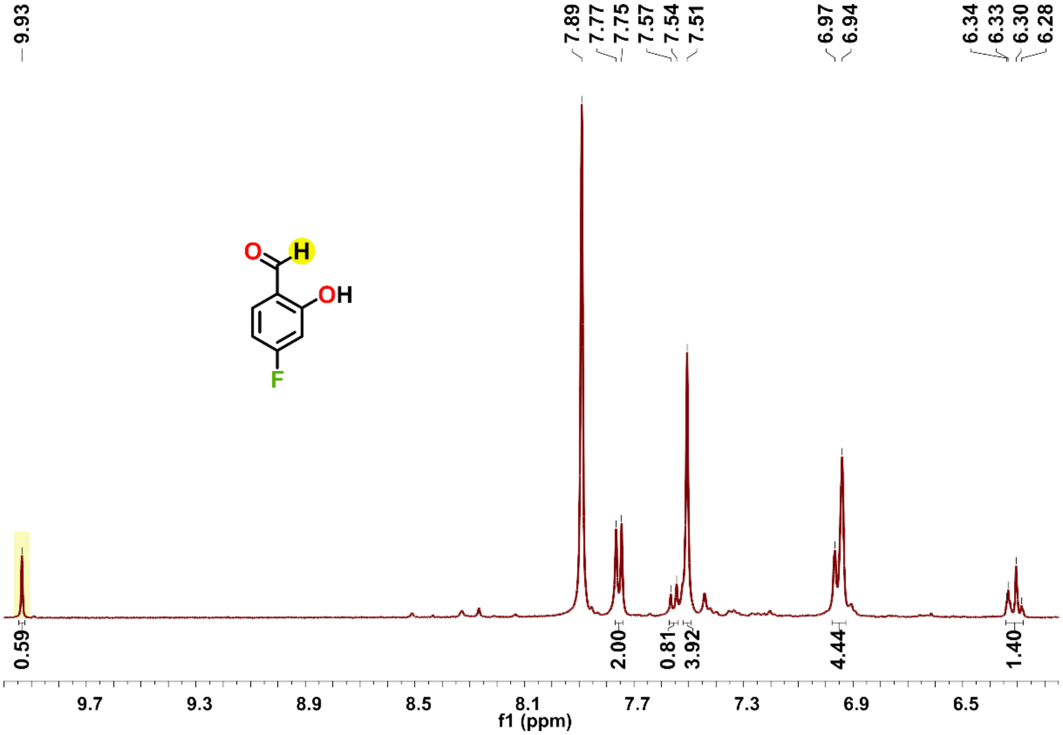

Figure S26. ^{1}H NMR of **1-A** in K_3PO_4/D_2O (aromatic and aldehydic proton regions; peak at δ = 7.89 corresponds to the aldehyde proton in DMF).

Table S3. ^{1}H NMR Peaks of Digested **1-A** for Quantification

**Linker Peaks**	**Position (ppm)**	**Integration**	**Protons Per Molecule**	**Normalization Value**
Red	7.76	2.00	2	1.00
Orange	6.96	4.44	4	*
Pink	7.51	3.92	4	0.98
			**Average**	**0.99**
**Aldehyde Peaks**				
Green	6.68	1.40	2	0.70
Blue	7.38	0.81	1	*
Yellow	10.05	0.59	1	0.59
			**Average**	**0.65**

Linker to aldehyde ratio = 0.99:0.65 = 1.52:1.00

-NH_2 groups to aldehyde = 3.05:1.00 = **33% of amino groups are modified**

*These peaks are overlapping with other peaks and were not used for quantification

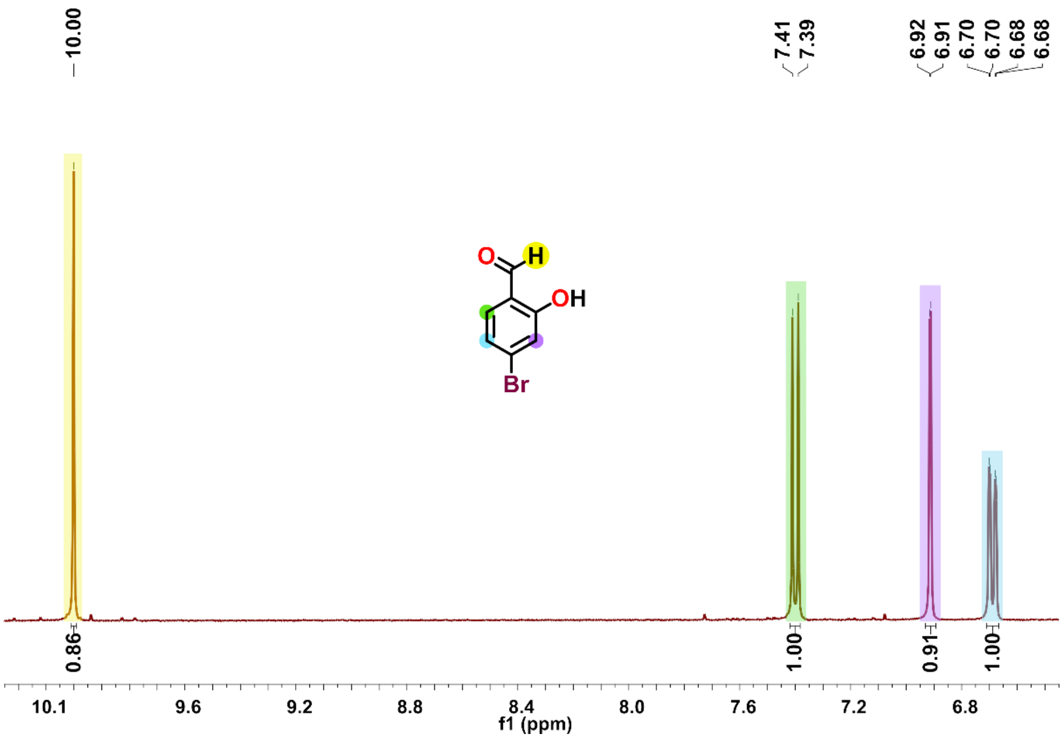

Figure S27. ^{1}H NMR (aromatic and aldehydic proton region) of 4-bromosalicylaldehyde in K_3PO_4/D_2O.

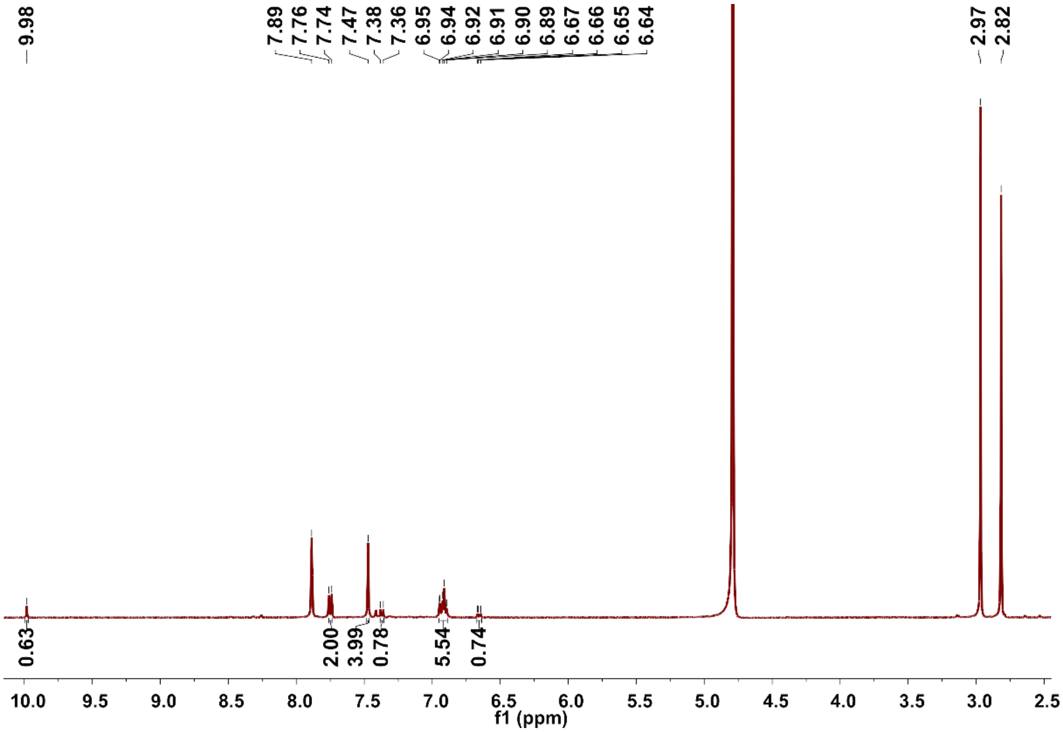

Figure S28. ^{1}H NMR of **1-B** in K_3PO_4/D_2O (peaks at δ = 2.82, 2.97, and 7.89 correspond to the protons in DMF).

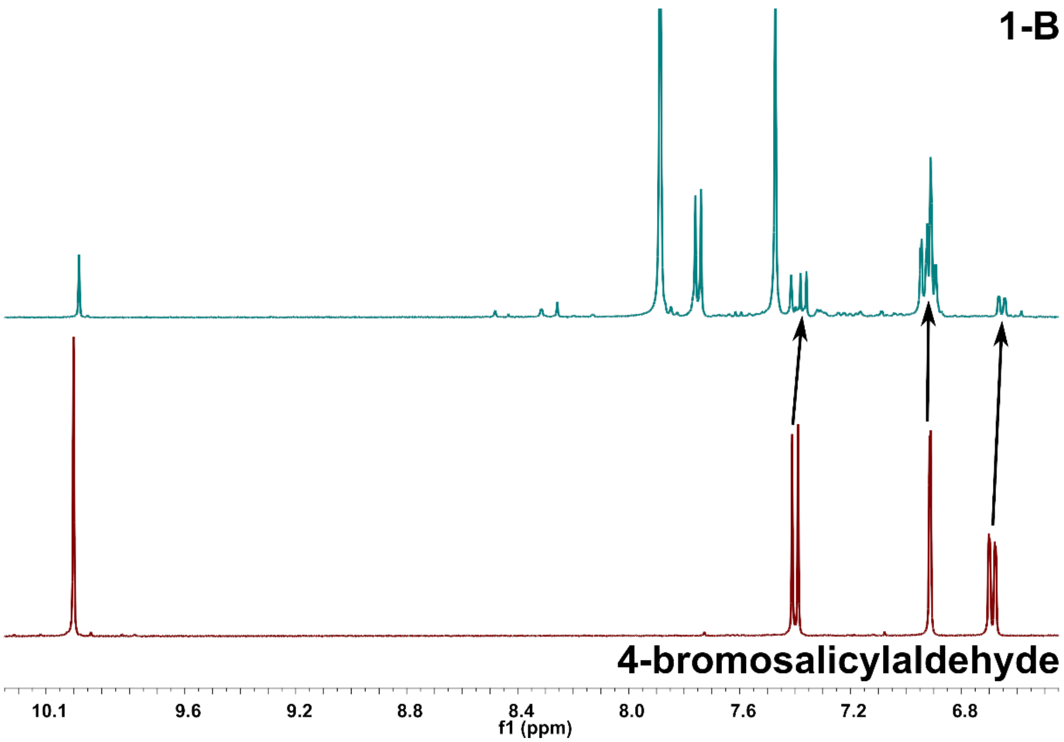

Figure S29. Overlay of ^{1}H NMR (aromatic and aldehydic proton region) of 4-bromosalicylaldehyde (bottom) and **1-B** (top).

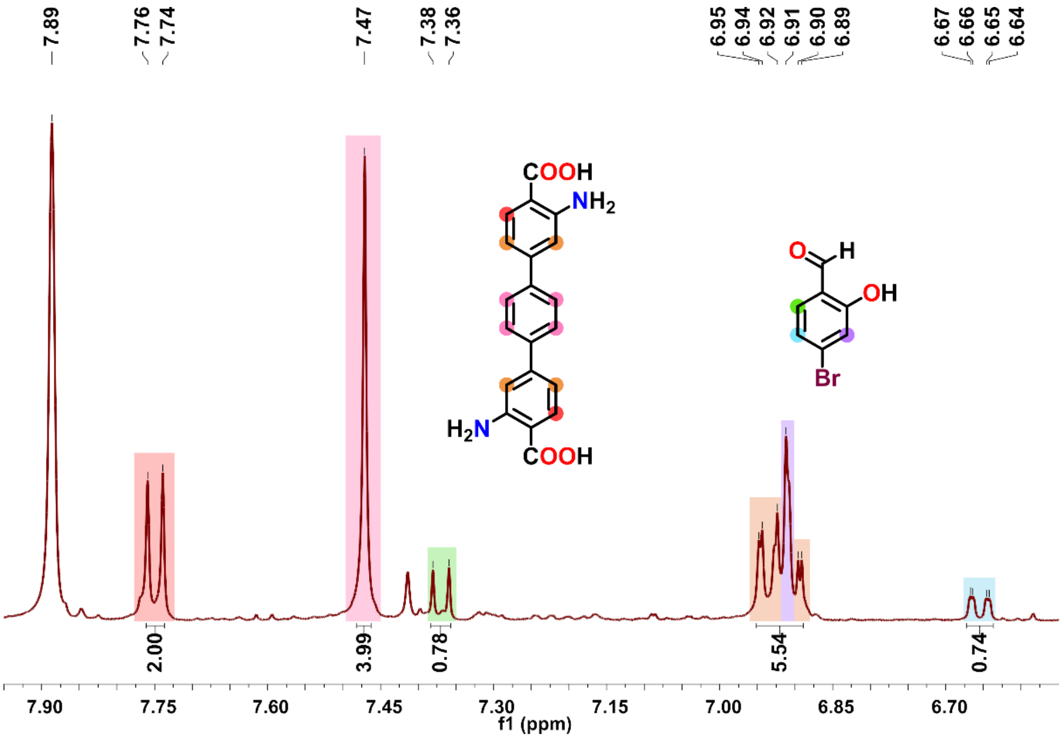

Figure S30. ^{1}H NMR of **1-B** in K_3PO_4/D_2O (aromatic region; peak at δ = 7.89 corresponds to the aldehyde proton in DMF).

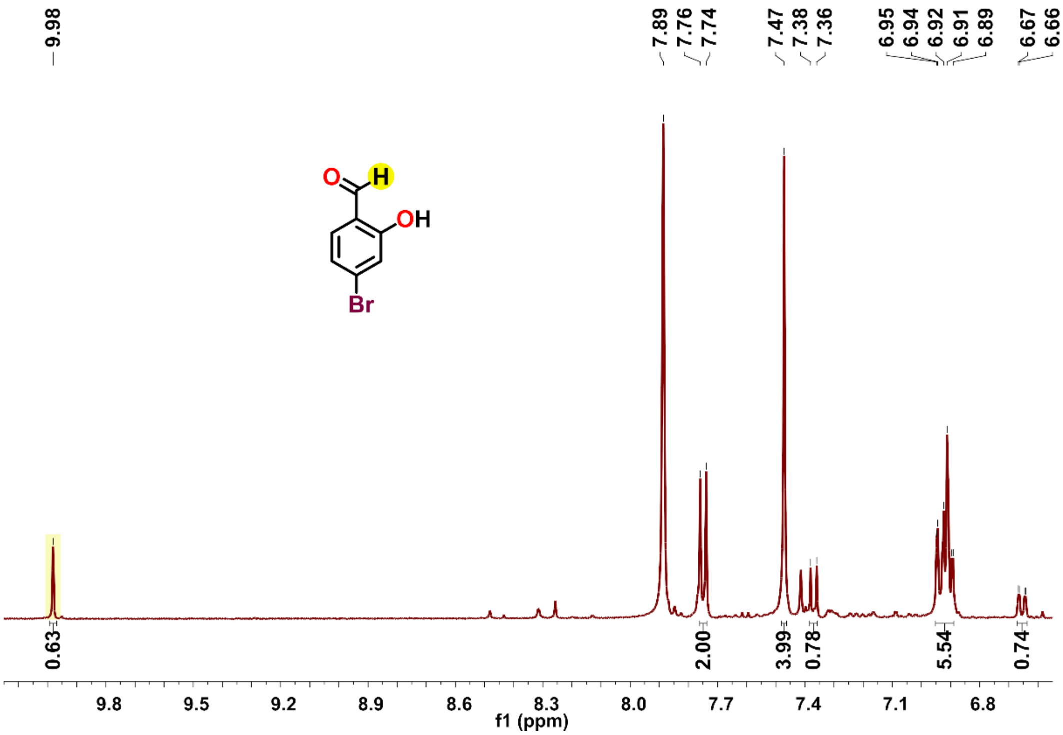

Figure S31. ^{1}H NMR of **1-B** in K_3PO_4/D_2O (aromatic and aldehydic proton regions; peak at δ = 7.89 corresponds to the aldehyde protons in DMF).

Table S4. ^{1}H NMR Peaks of Digested **1-B** for Quantification

**Linker Peaks**	**Position (ppm)**	**Integration**	**Protons Per Molecule**	**Normalization Value**
Red	7.75	2.00	2	1.00
Orange	6.95-6.89	--	4	*
Pink	7.47	3.99	4	1.00
			**Average**	**1.00**
**Aldehyde Peaks**				
Green	7.35	0.78	1	0.78
Blue	6.65	0.74	1	0.74
Purple	6.91	--	1	*
Yellow	9.98	0.63	1	0.63
			**Average**	**0.72**

Linker to aldehyde ratio = 1.00:0.72 = 1.39:1.00

-NH_2 groups to aldehyde = 2.78:1.00 = **36% of amino groups are modified**

*These peaks are overlapping with other peaks and were not used for quantification

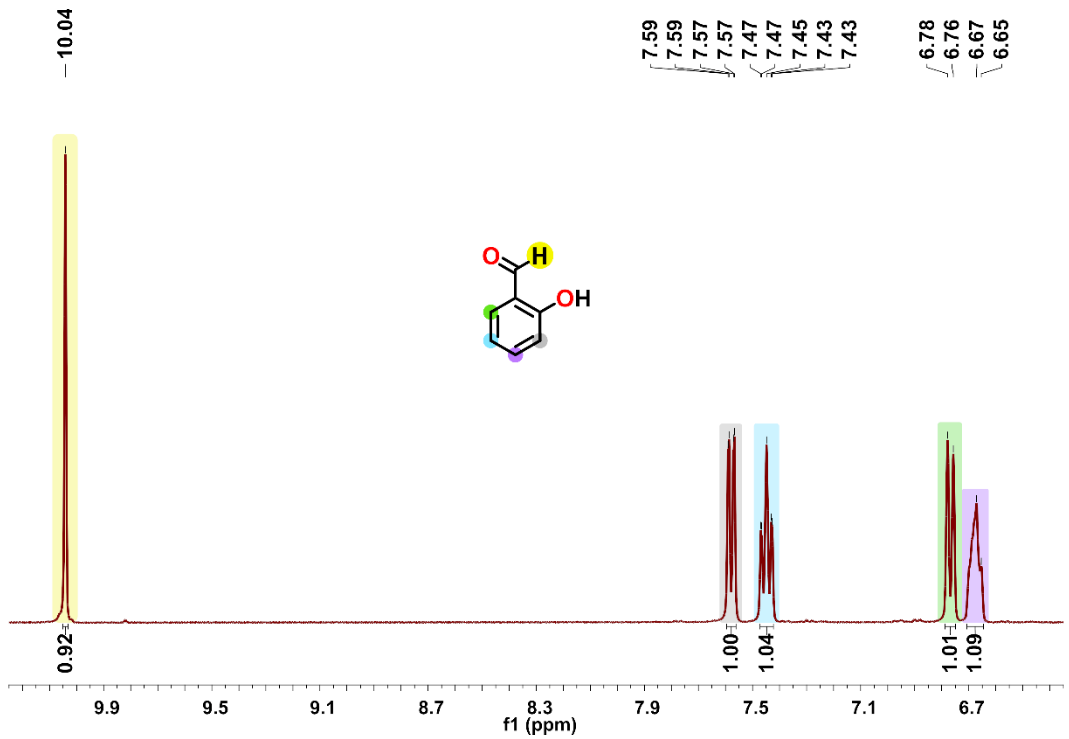

Figure S32. ^{1}H NMR (aromatic and aldehydic proton region) of salicylaldehyde in K_3PO_4/D_2O.

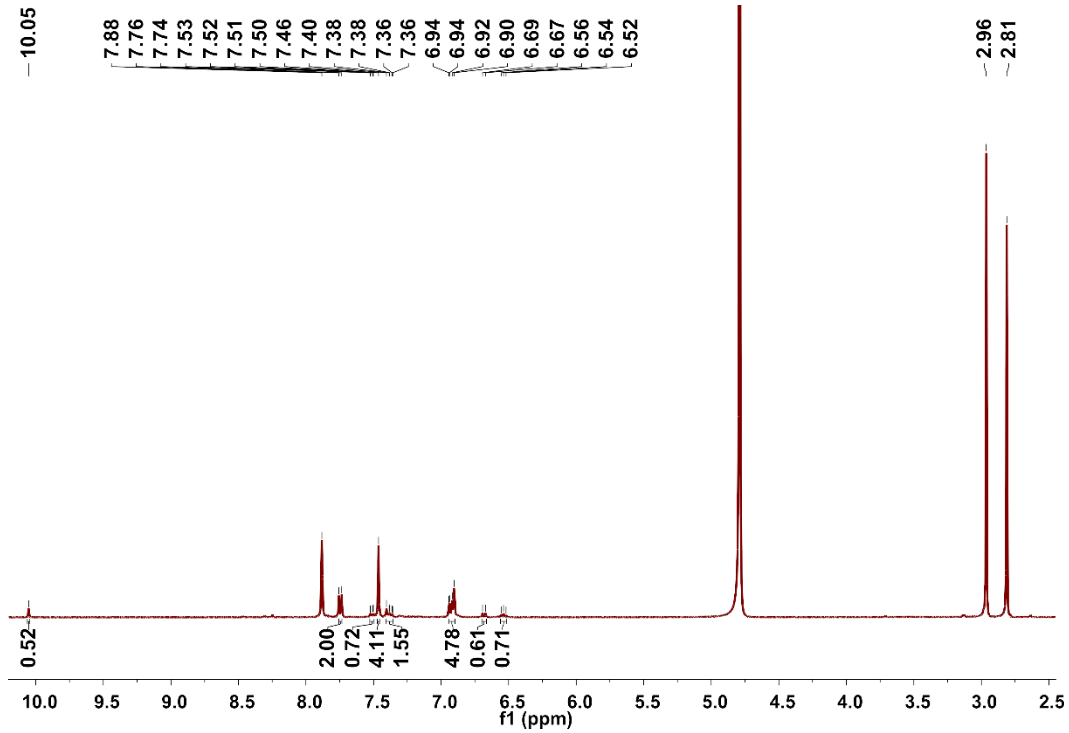

Figure S33. ^{1}H NMR of **1-C** in K_3PO_4/D_2O (peaks at δ = 2.81, 2.96, and 7.81 correspond to the protons in DMF).

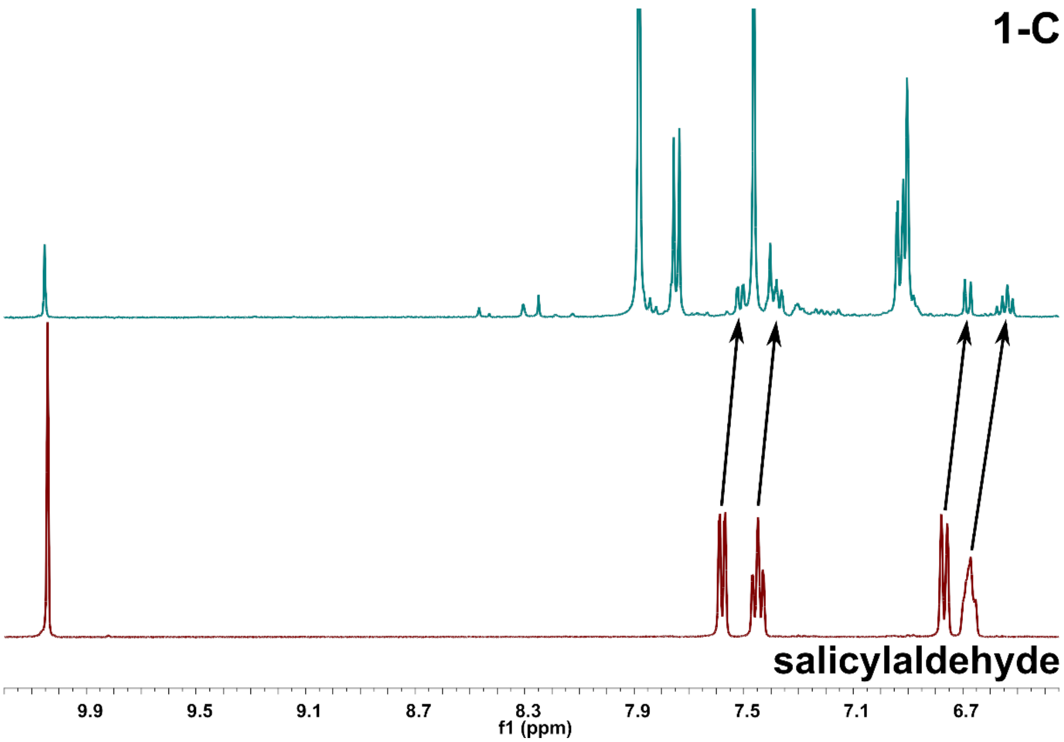

Figure S34. Overlay of ^{1}H NMR (aromatic and aldehydic proton region) of salicylaldehyde (bottom) and **1-C** (top).

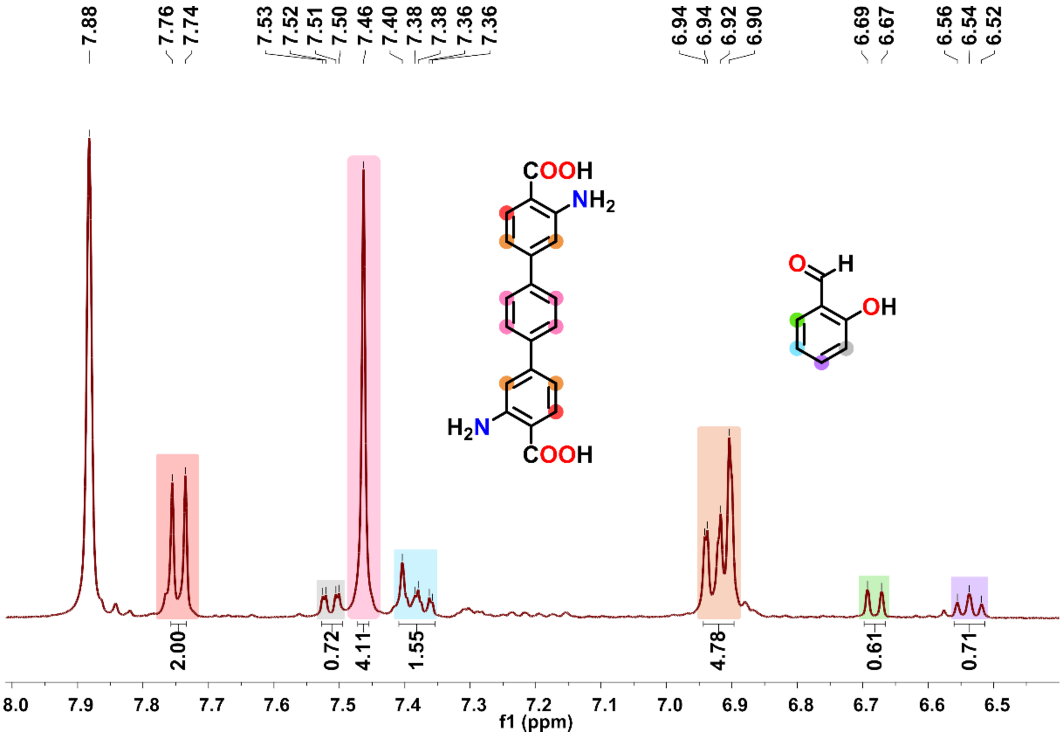

Figure S35. ^{1}H NMR of **1-C** in K_3PO_4/D_2O (aromatic region; peak at δ = 7.88 corresponds to the aldehyde proton in DMF).

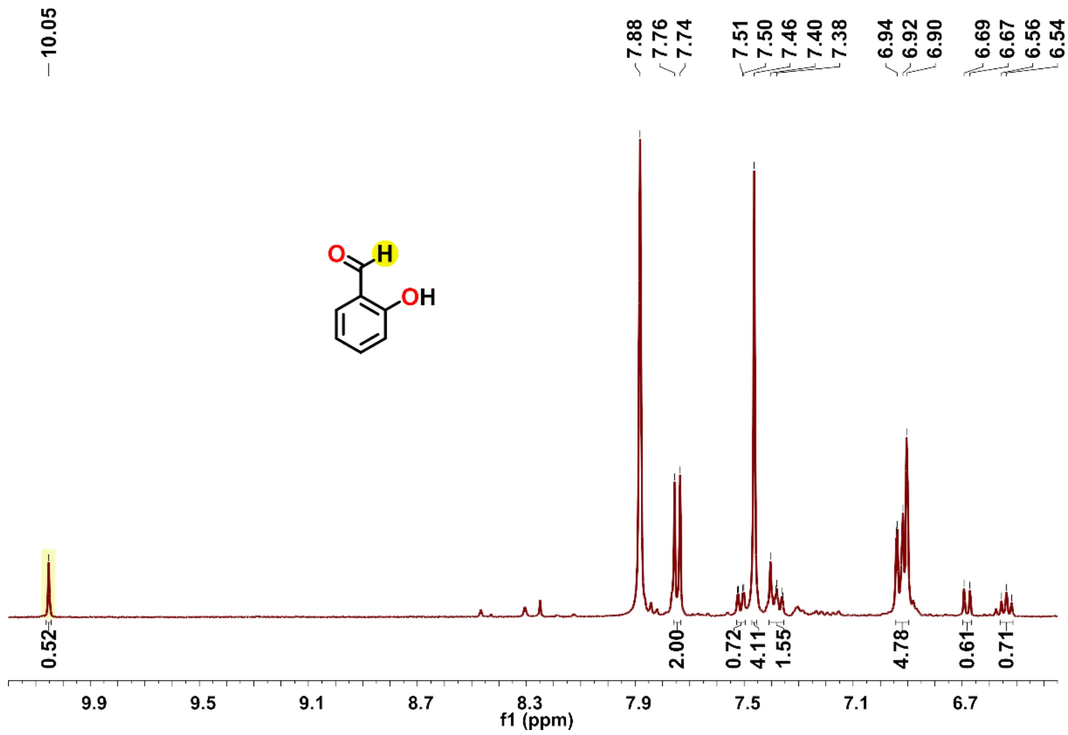

Figure S36. ^{1}H NMR of **1-C** in K_3PO_4/D_2O (aromatic and aldehydic proton regions; peak at δ = 7.88 corresponds to the aldehyde proton in DMF).

Table S5. ^{1}H NMR Peaks of Digested **1-C** for Quantification

Linker Peaks	Position (ppm)	Integration	Protons Per Molecule	Normalization Value
Red	7.75	2.00	2	1.00
Orange	6.91	4.78	4	*
Pink	7.46	4.11	4	1.03
			**Average**	**1.02**
**Aldehyde Peaks**				
Green	6.68	0.61	1	0.61
Blue	7.38	1.55	1	*
Purple	6.54	0.71	1	0.71
Grey	7.51	0.72	1	0.72
Yellow	10.05	0.52	1	0.52
			**Average**	**0.64**

Linker to aldehyde ratio = 1.02:0.64 = 1.59:1.00

-NH_2 groups to aldehyde = 3.18:1.00 = **31% of amino groups are modified**

*These peaks are overlapping with other peaks and were not used for quantification

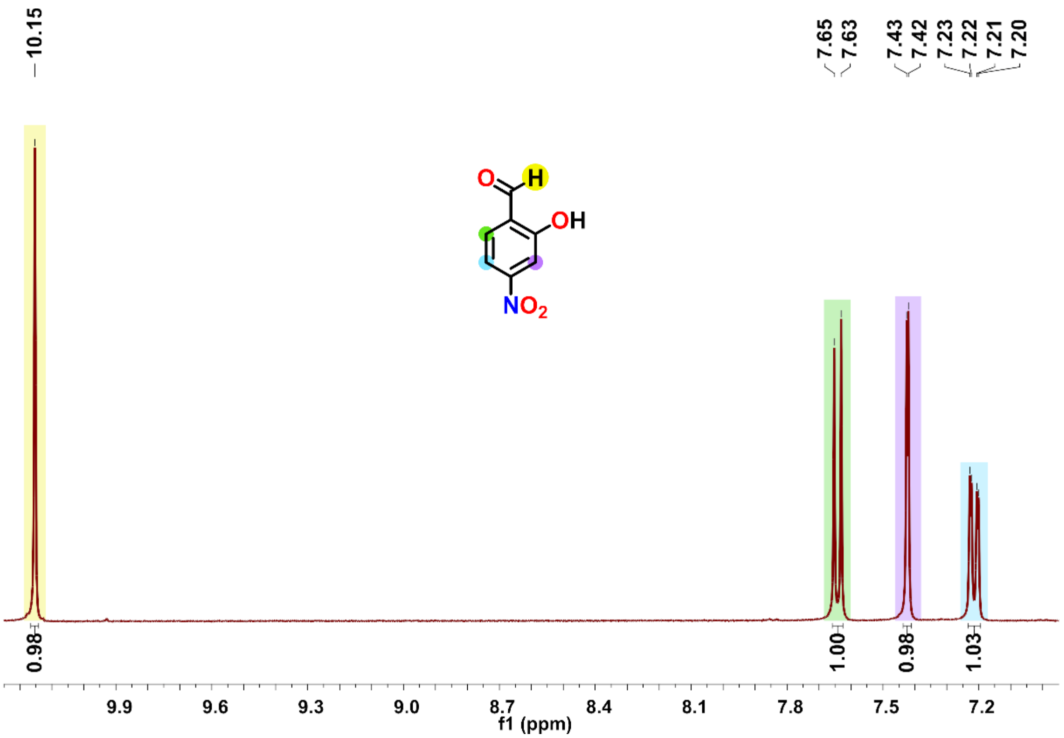

Figure S37. ^{1}H NMR (aromatic and aldehydic proton region) of 4-nitrosalicylaldehyde in K_3PO_4/D_2O.

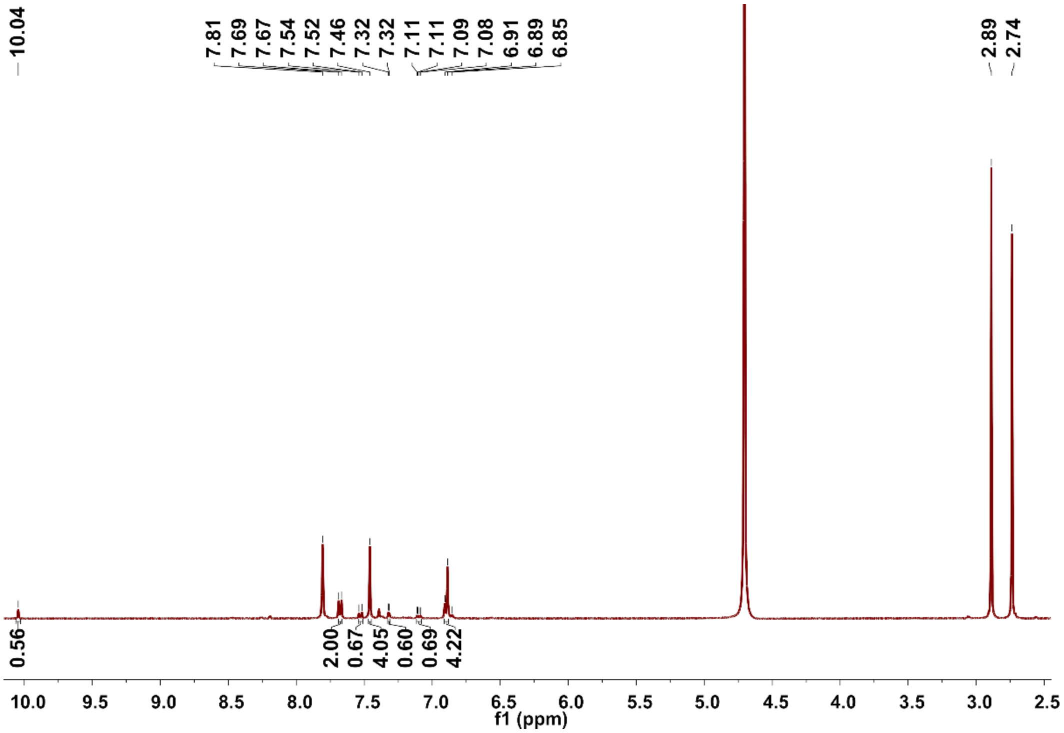

Figure S38. ^{1}H NMR of **1-D** in K_3PO_4/D_2O (peaks at δ = 2.74, 2.89, and 7.81 correspond to the protons in DMF).

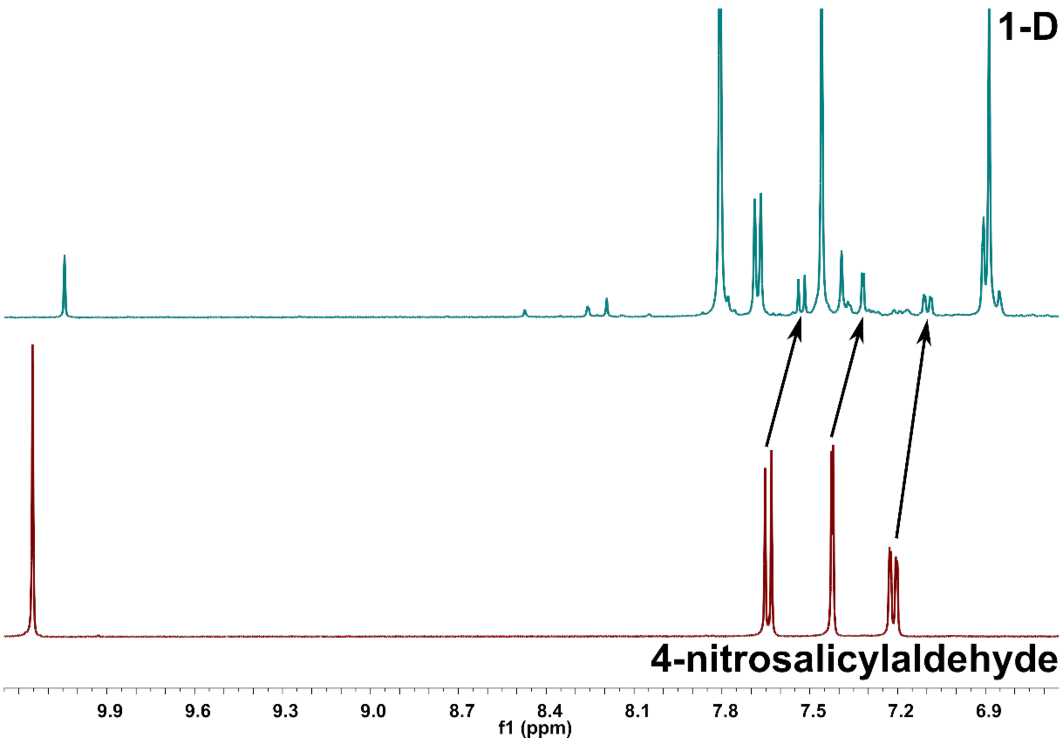

Figure S39. Overlay of ^{1}H NMR (aromatic and aldehydic proton region) of 4-nitrosalicylaldehyde (bottom) and **1-D** (top).

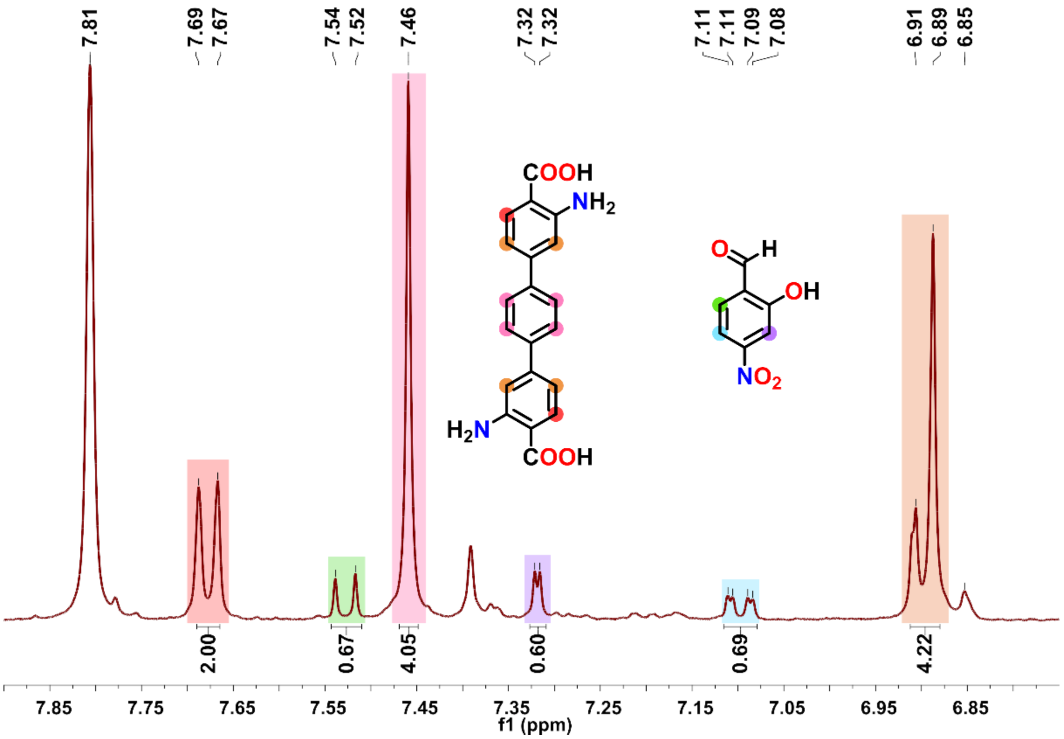

Figure S40. ^{1}H NMR of **1-D** in K_3PO_4/D_2O (aromatic region; peak at δ = 7.81 corresponds to the aldehyde proton in DMF).

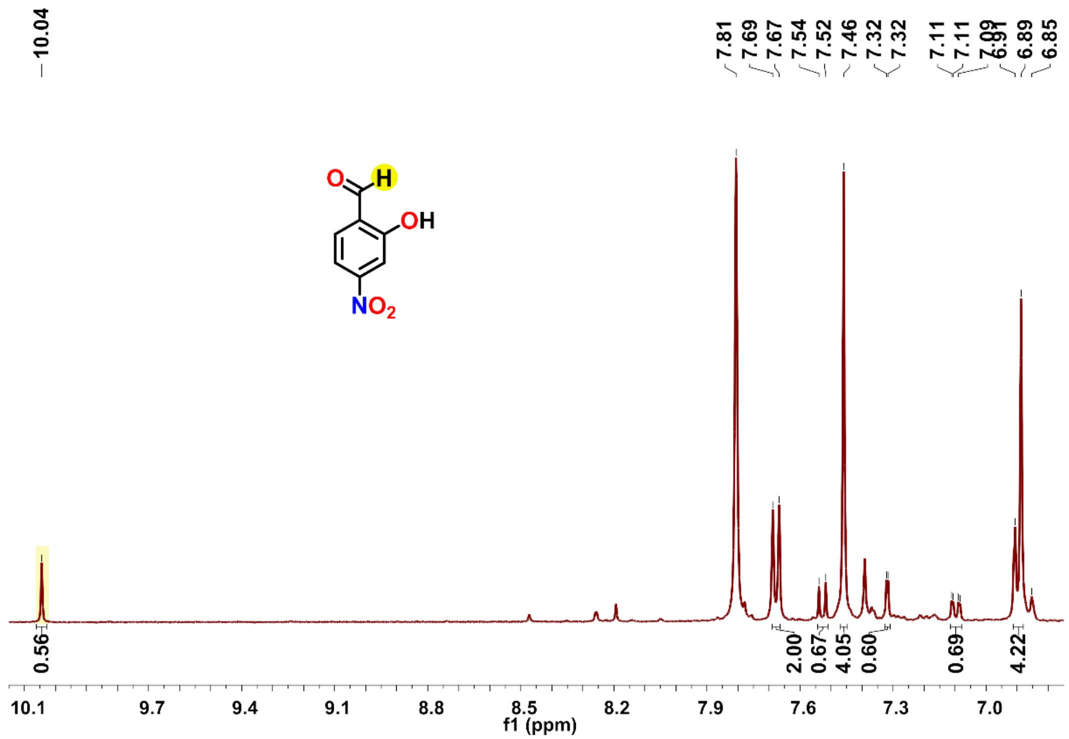

Figure S41. ^{1}H NMR of **1-D** in K_3PO_4/D_2O (aromatic and aldehydic proton regions; peaks at δ = 7.81 corresponds to the aldehyde protons in DMF).

Table S6. ^{1}H NMR Peaks of Digested **1-D** for Quantification

Linker Peaks	Position (ppm)	Integration	Protons Per Molecule	Normalization Value
Red	7.68	2.00	2	1.00
Orange	6.89	4.22	4	*
Pink	7.46	4.05	4	1.01
			**Average**	**1.00**
**Aldehyde Peaks**				
Green	7.53	0.67	1	0.67
Blue	7.10	0.69	1	0.69
Purple	7.32	0.60	1	0.60
Yellow	10.04	0.56	1	0.56
			**Average**	**0.63**

Linker to aldehyde ratio = 1.00:0.63 = 1.59:1.00

-NH_2 groups to aldehyde = 3.18:1.00 = **31% of amino groups are modified**

*These peaks are overlapping with other peaks and were not used for quantification

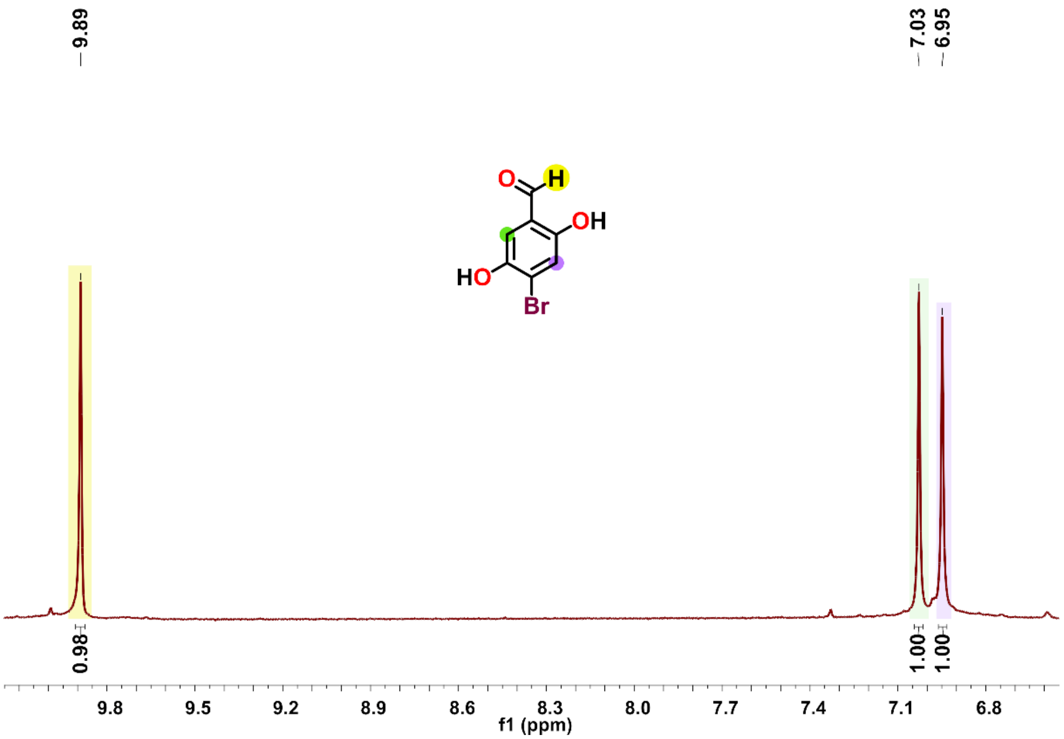

Figure S42. ^{1}H NMR (aromatic and aldehydic proton region) of 2,5-dihydroxy-4-bromobenzaldehyde in K_3PO_4/D_2O.

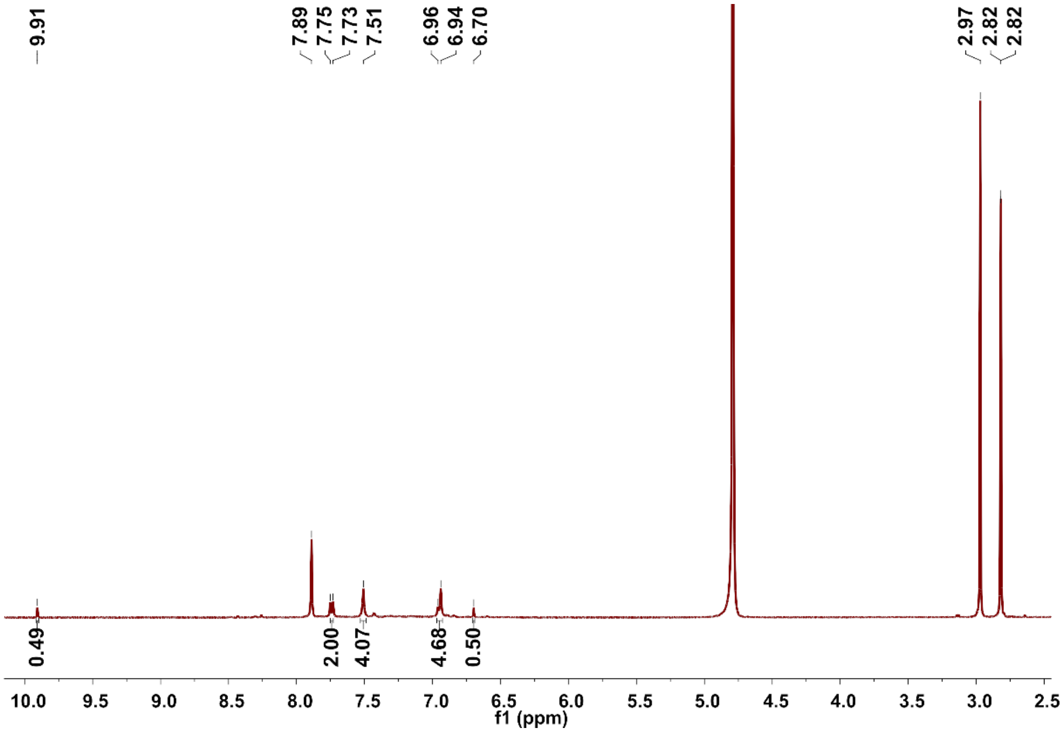

Figure S43. ^{1}H NMR of **1-E** in K_3PO_4/D_2O (peaks at δ = 2.82, 2.97, and 7.89 correspond to the protons in DMF).

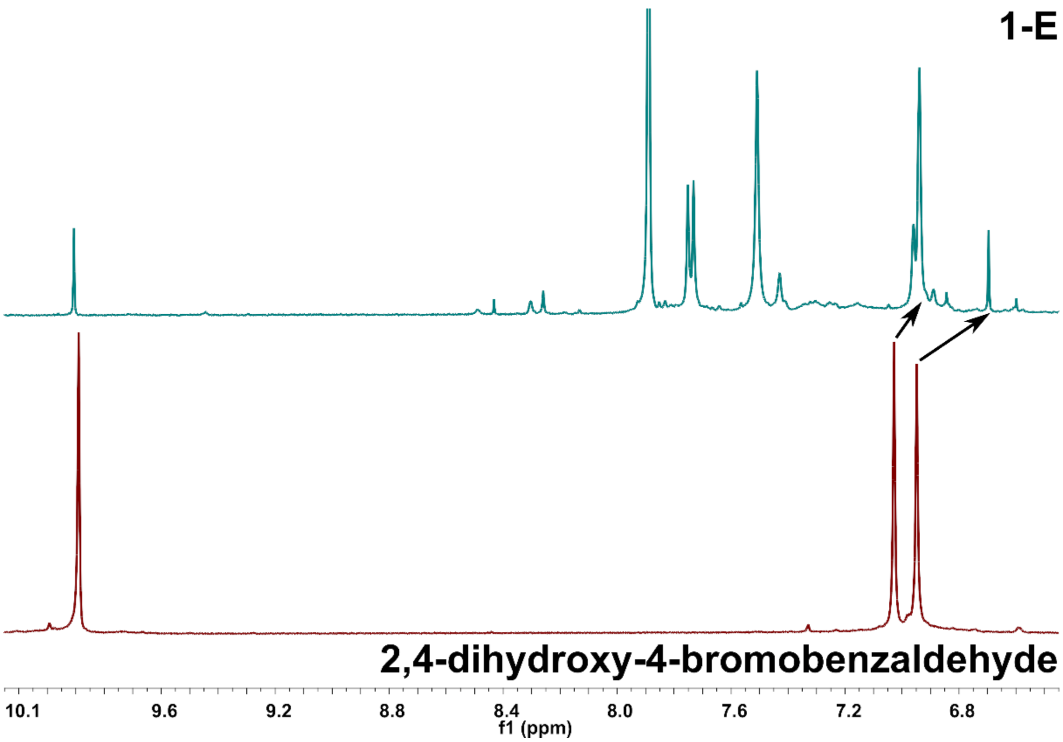

Figure S44. Overlay of ^{1}H NMR (aromatic and aldehydic proton region) of 2,5-dihydroxy-4-bromobenzaldehyde (bottom) and **1-E** (top).

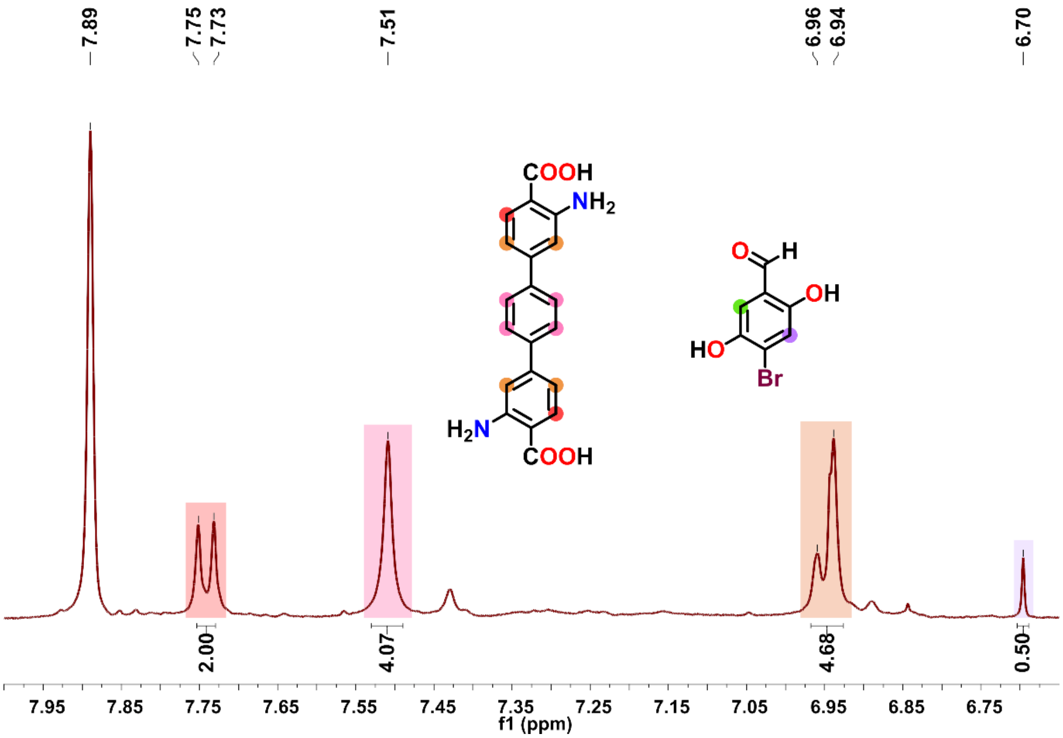

Figure S45. ^{1}H NMR of **1-E** in K_3PO_4/D_2O (aromatic region; peaks at δ = 7.89 corresponds to the aldehyde proton in DMF).

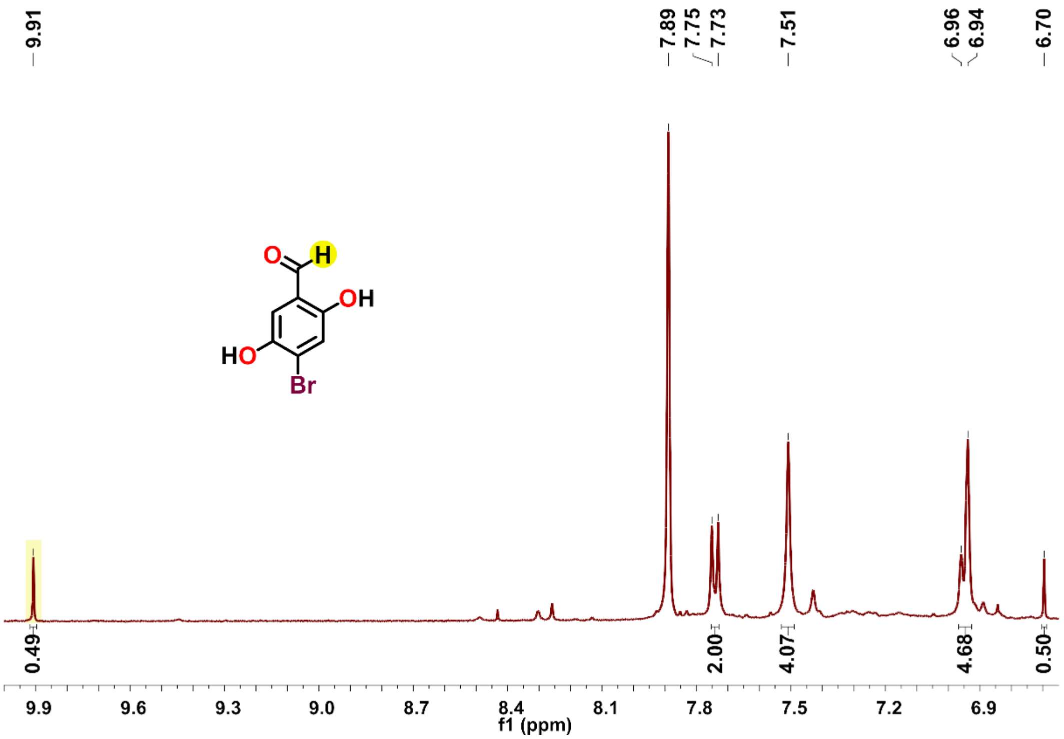

Figure S46. ^1H NMR of **1-E** in K_3PO_4/D_2O (aromatic and aldehydic proton regions; peaks at δ = 7.89 corresponds to the aldehyde protons in DMF)

Table S7. ^1H NMR Peaks of Digested **1-E** for Quantification

Linker Peaks	Position (ppm)	Integration	Protons Per Molecule	Normalization Value
Red	7.74	2.00	2	1.00
Orange	6.95	4.68	4	*
Pink	7.51	4.07	4	1.04
			**Average**	**1.02**
**Aldehyde Peaks**				
Green	6.95	*	1	*
Purple	6.70	0.50	1	0.50
Yellow	9.91	0.49	1	0.49
			**Average**	**0.50**

Linker to aldehyde ratio = 1.00:0.50 = 2.00:1.00

-NH_2 groups to aldehyde = 4.00:1.00 = **25% of amino groups are modified**

*These peaks are overlapping with other peaks and were not used for quantification

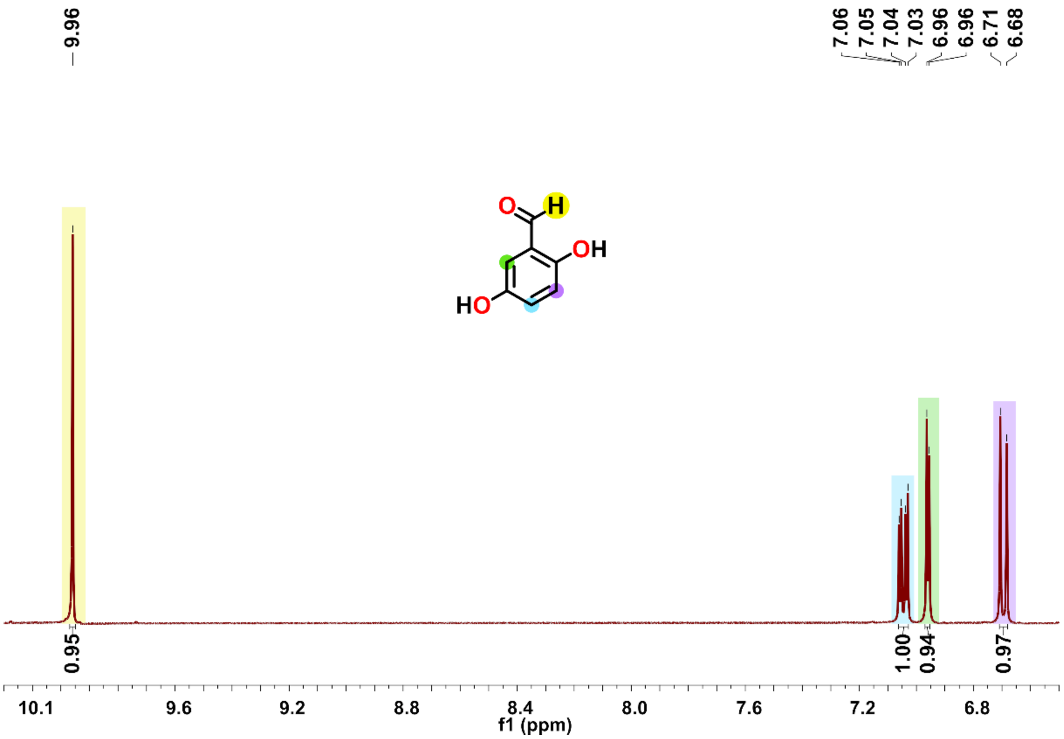

Figure S47. ^{1}H NMR (aromatic and aldehydic proton region) of 2,5-dihydroxybenzaldehyde in K_3PO_4/D_2O.

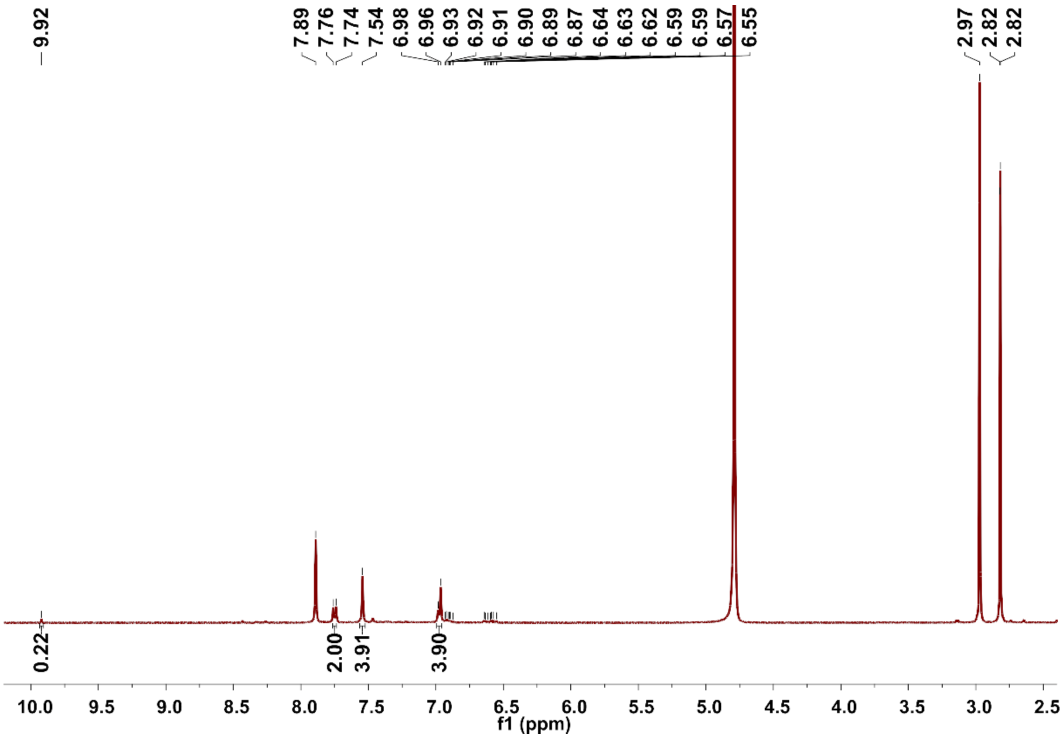

Figure S48. ^{1}H NMR of **1-F** in K_3PO_4/D_2O (peaks at δ = 2.82, 2.97, and 7.89 correspond to the protons in DMF).

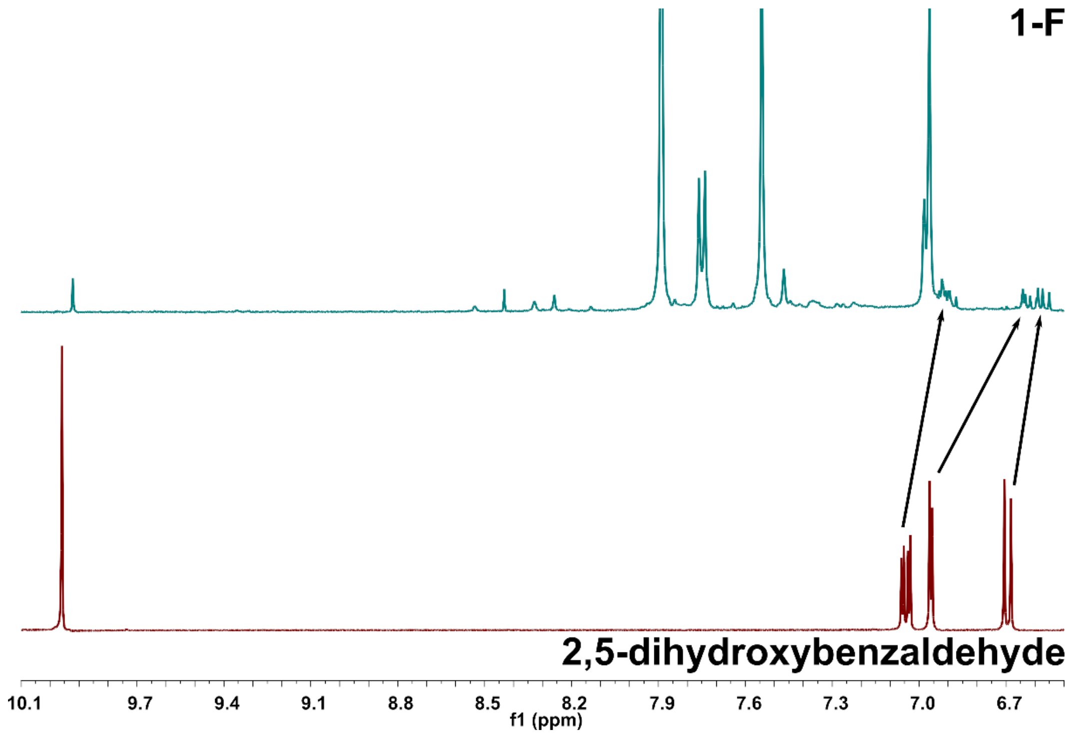

Figure S49. Overlay of ^{1}H NMR (aromatic and aldehydic proton region) of 2,5-dihydroxybenzaldehyde (bottom) and **1-F** (top).

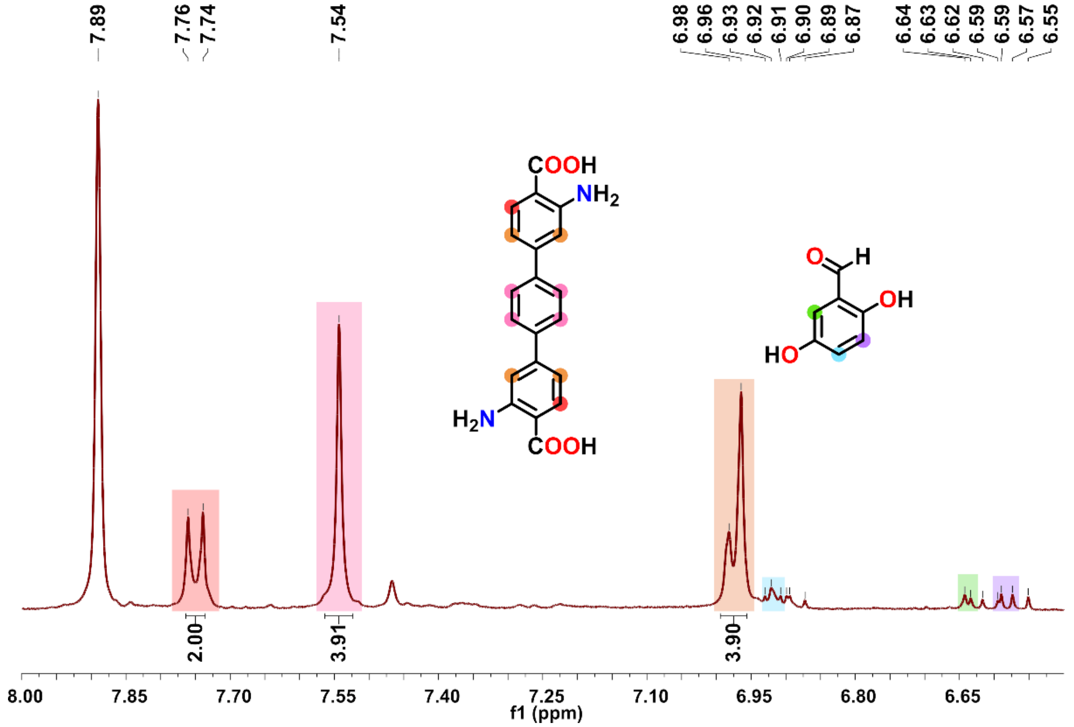

Figure S50. ^{1}H NMR of **1-F** in K_3PO_4/D_2O (aromatic region; peak at δ = 7.89 corresponds to the aldehyde protons in DMF).

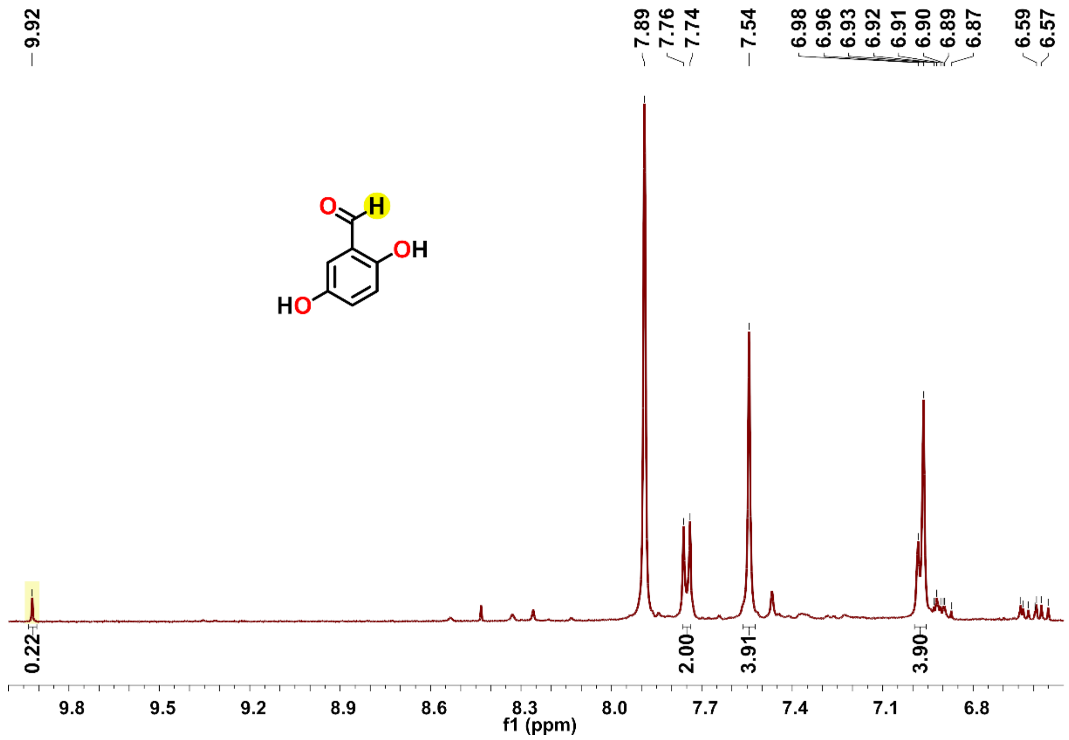

Figure S51. ^{1}H NMR of **1-F** in K_3PO_4/D_2O (aromatic and aldehydic proton regions; peaks at δ = 7.89 corresponds to the aldehyde protons in DMF)

Table S8. ^{1}H NMR Peaks of Digested **1-F** for Quantification

Linker Peaks	Position (ppm)	Integration	Protons Per Molecule	Normalization Value
Red	7.75	2.00	2	1.00
Orange	6.93	4.69	4	*
Pink	7.49	4.00	4	1.00
			**Average**	**1.00**
**Aldehyde Peaks**				
Green	6.63	*	1	*
Blue	6.89	*	1	*
Purple	6.57	*	1	*
Yellow	9.92	0.22	1	0.22
			**Average**	**0.22**

Linker to aldehyde ratio = 1.00:0.22 = 4.55:1.00

$-NH_2$ groups to aldehyde = 9.09:1.00 = **11% of amino groups are modified**

*These peaks are overlapping with other peaks and were not used for quantification

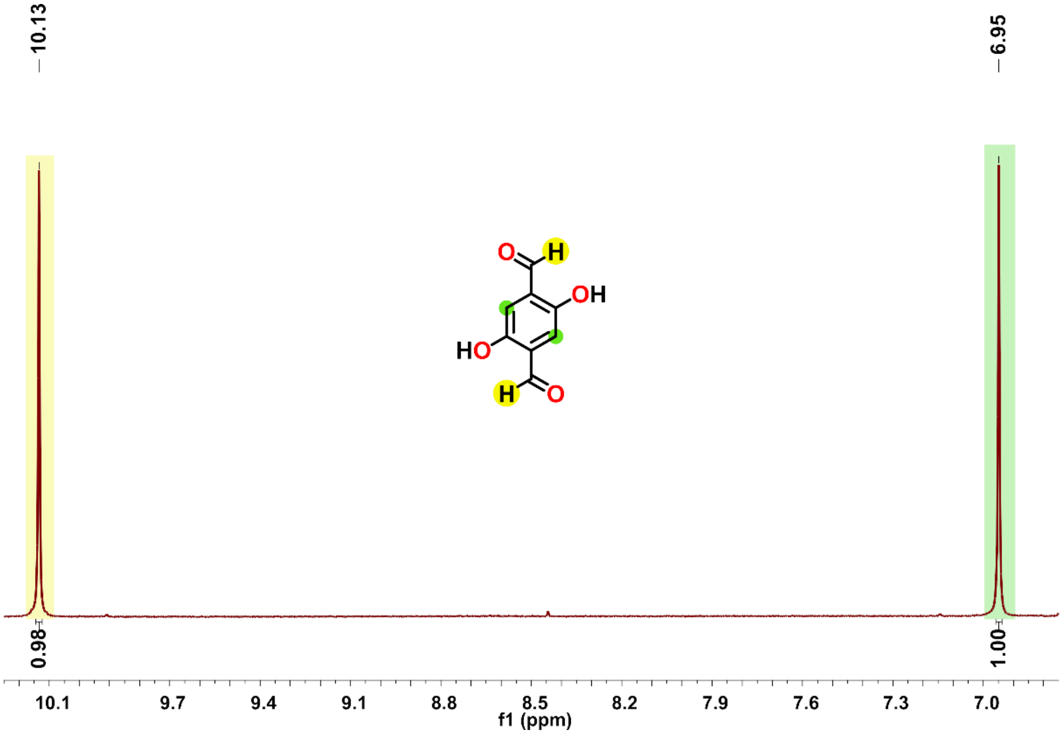

Figure S52. ^{1}H NMR (aromatic and aldehydic proton region) of 2,5-dihydroxyterephthaldehyde in K_3PO_4/D_2O.

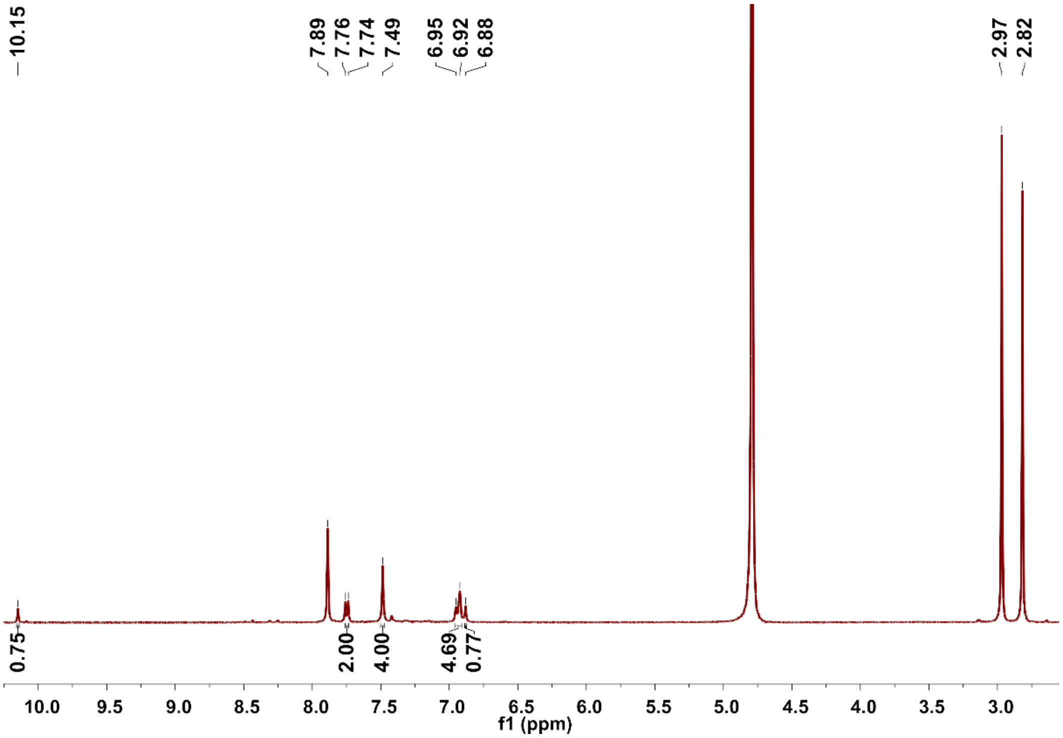

Figure S53. ^{1}H NMR of **1-G** in K_3PO_4/D_2O (peaks at δ = 2.82, 2.97, and 7.89 correspond to the protons in DMF).

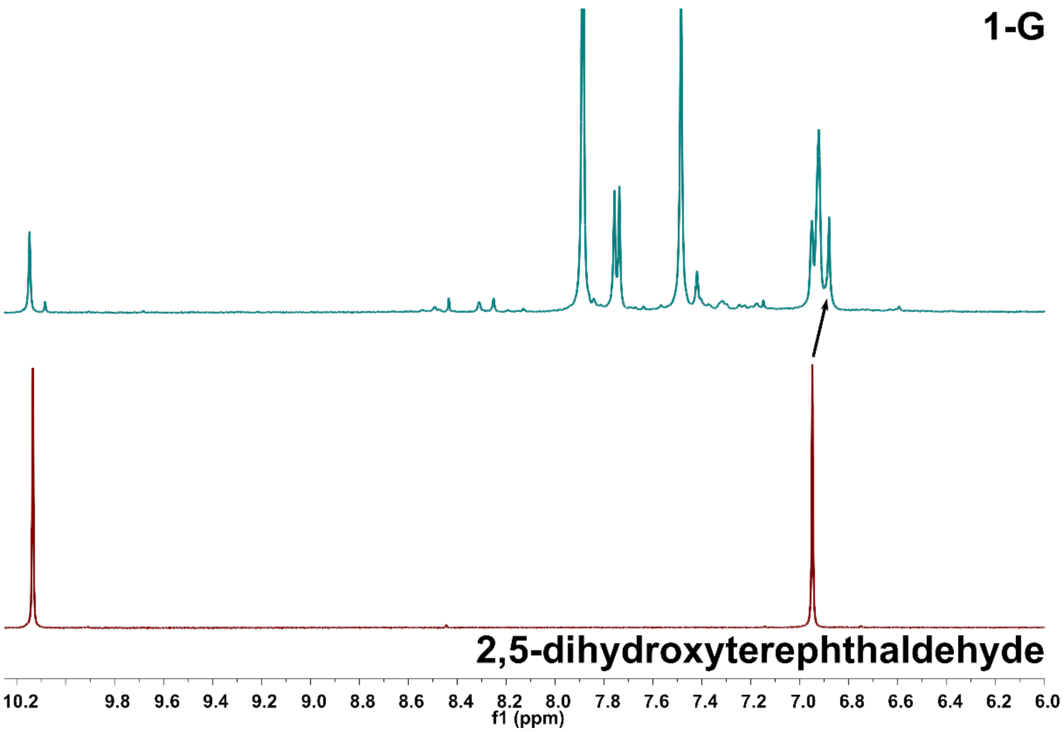

Figure S54. Overlay of ^{1}H NMR (aromatic and aldehydic proton region) of 2,5-dihydroxyterephthaldehyde (bottom) and **1-G** (top).

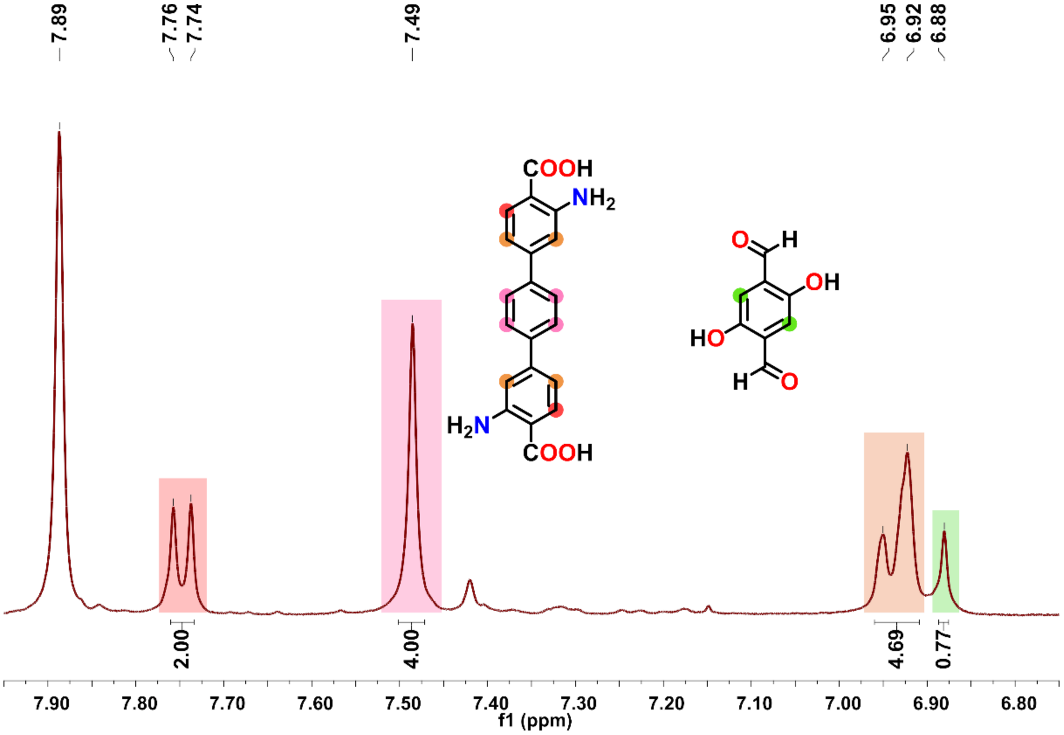

Figure S55. ^{1}H NMR of **1-G** in K_3PO_4/D_2O (aromatic region; peak at δ = 7.89 corresponds to the aldehyde protons in DMF).

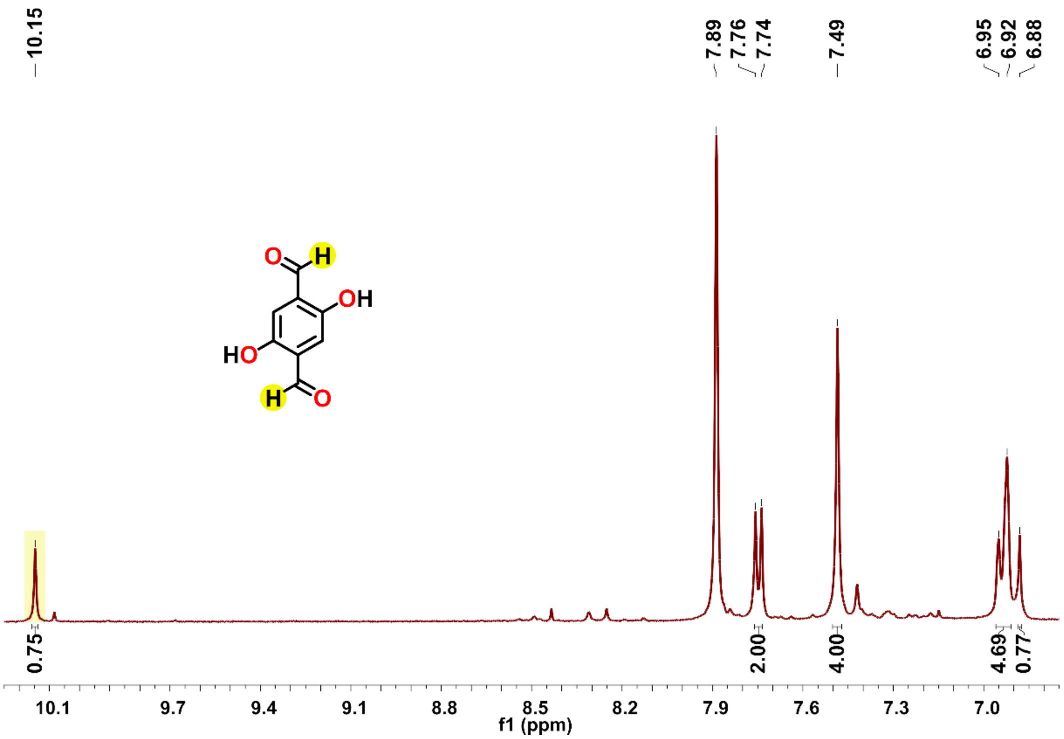

Figure S56. ^{1}H NMR of **1-G** in K_3PO_4/D_2O (aromatic and aldehydic proton regions; peaks at δ = 7.89 corresponds to the aldehyde protons in DMF).

Table S9. ^{1}H NMR Peaks of Digested **1-G** for Quantification

Linker Peaks	Position (ppm)	Integration	Protons Per Molecule	Normalization Value
Red	7.75	2.00	2	1.00
Orange	6.93	4.69	4	*
Pink	7.49	4.00	4	1.00
			**Average**	**1.00**
**Aldehyde Peaks**				
Green	6.88	0.77	1	0.77
Yellow	10.15	0.75	1	0.75
			**Average**	**0.76**

Linker to aldehyde ratio = 1.00:0.76 = 1.32:1.00

-NH_2 groups to aldehyde = 2.63:1.00 = **38% of amino groups are modified**

*These peaks are overlapping with other peaks and were not used for quantification

4.4 UV-Vis Absorption

Table S10. Fitted UV-Vis absorption peak positions and peak widths of all MOFs.

MOF	Backbone Peak Position (nm)	Amino Functionalized Phenyl Ring Peak Position (nm)	Extended π Conjugation Peak Position (nm)
1	308.1	370.0	N/A
1-A	311.6	375.0	430.7
1-B	315.0	383.7	447.8
1-C	316.1	378.7	450.5
1-D	301.8	379.2	479.3
1-E	313.8	376.8	495.9
1-F	314.3	377.9	506.5
1-G	310.1	378.8	535.0
**MOF**	**Backbone Peak Width (nm)**	**Amino Functionalized Phenyl Ring Peak Width (nm)**	**Extended π Conjugation Peak Width (nm)**
1	60.0	53.5	N/A
1-A	85.9	48.6	72.8
1-B	107.0	49.4	64.0
1-C	76.0	46.8	72.9
1-D	154.0	42.1	81.4
1-E	138.2	48.9	96.6
1-F	132.1	45.2	105.5
1-G	140.0	41.8	139.2

5. References

1. Schaate, A.; Roy, P.; Godt, A.; Lippke, J.; Waltz, F.; Wiebcke, M.; Behrens, P., Modulated Synthesis of Zr-Based Metal-Organic Frameworks: From Nano to Single Crystals. *Chem. Eur. J.* **2011**, *17*, 6643-6651.
2. Ma, J.; Kalenak, A. P.; Wong-Foy, A. G.; Matzger, A. J., Rapid Guest Exchange and Ultra-Low Surface Tension Solvents Optimize Metal–Organic Framework Activation. *Angew. Chem.* **2017**, *129*, 14810-14813.
3. Dubbeldam, D.; Calero, S.; Ellis, D. E.; Snurr, R. Q., RASPA: molecular simulation software for adsorption and diffusion in flexible nanoporous materials. *Molecular Simulation* **2015**, *42*, 81-101.
4. Potoff, J. J.; Siepmann, J. I., Vapor–liquid equilibria of mixtures containing alkanes, carbon dioxide, and nitrogen. *AIChE J.* **2001**, *47*, 1676-1682.
5. Manz, T. A.; Limas, N. G., Introducing DDEC6 atomic population analysis: part 1. Charge partitioning theory and methodology. *RSC Adv.* **2016,** *6* (53), 47771-47801.
6. Limas, N. G.; Manz, T. A., Introducing DDEC6 atomic population analysis: part 2. Computed results for a wide range of periodic and nonperiodic materials. *RSC Adv.* **2016**, *6*, 45727-45747.
7. Brunauer, S.; Emmett, P. H.; Teller, E., Adsorption of Gases in Multimolecular Layers. *J. Am. Chem. Soc.* **1938**, *60*, 309-319.
8. Wang, T. C.; Bury, W.; Gomez-Gualdron, D. A.; Vermeulen, N. A.; Mondloch, J. E.; Deria, P.; Zhang, K.; Moghadam, P. Z.; Sarjeant, A. A.; Snurr, R. Q.; Stoddart, J. F.; Hupp, J. T.; Farha, O. K., Ultrahigh Surface Area Zirconium MOFs and Insights into the Applicability of the BET Theory. *J. Am. Chem. Soc.* **2015**, *137*, 3585-3591.
9. Gomez-Gualdron, D. A.; Moghadam, P. Z.; Hupp, J. T.; Farha, O. K.; Snurr, R. Q., Application of Consistency Criteria To Calculate BET Areas of Micro- And Mesoporous Metal-Organic Frameworks. *J. Am. Chem. Soc.* **2016**, *138*, 215-224.
10. Bredas, J.-L., Mind the gap! *Mater. Horiz.* **2014**, *1*, 17-19.
11. Kimber, P.; Plasser, F., Energy Component Analysis for Electronically Excited States of Molecules: Why the Lowest Excited State Is Not Always the HOMO/LUMO Transition. *J. Chem. Theory Comput.* **2023**, *19*, 2340-2352.
12. Gaussian 16, Revision C.01, M. J. Frisch, G. W. T., H. B. Schlegel, G. E. Scuseria, M. A. Robb, J. R. Cheeseman, G. Scalmani, V. Barone, G. A. Petersson, H. Nakatsuji, *et al*., Gaussian, Inc., Wallingford CT, **2016**.
13. Grimme, S.; Antony, J.; Ehrlich, S.; Krieg, H., A consistent and accurate *ab initio* parametrization of density functional dispersion correction (DFT-D) for the 94 elements H-Pu. *J. Chem. Phys.* **2010**, *132*, 154104.
14. Frigo, M.; Johnson, S. G., The Design and Implementation of FFTW3. *Proceedings of the IEEE.* **2005**, *93*, 216-231.
15. VandeVondele, J.; Krack, M.; Mohamed, F.; Parrinello, M.; Chassaing, T.; Hutter, J., QUICKSTEP: Fast and accurate density functional calculations using a mixed Gaussian and plane waves approach. *Computer Physics Communications.* **2005**, *167*, 103-128.
16. VandeVondele, J.; Hutter, J., An efficient orbital transformation method for electronic structure calculations. *J. Chem. Phys.* **2003**, *118*, 4365-4369.
17. Iannuzzi, M.; Chassaing, T.; Wallman, T.; Hutter, J., Ground and Excited State Density Functional Calculations with the Gaussian and Augmented-Plane-Wave Method. *Chimia* **2005**, *59*, 499.
18. Martin, R. L., Natural transition orbitals. *J. Chem. Phys.* **2003**, *118*, 4775-4777.
19. Luo, T. Y.; Liu, C.; Eliseeva, S. V.; Muldoon, P. F.; Petoud, S.; Rosi, N. L., Rare Earth pcu Metal-Organic Framework Platform Based on $RE_4(\mu_3\text{-}OH)_4(COO)_6^{2+}$ Clusters: Rational

Design, Directed Synthesis, and Deliberate Tuning of Excitation Wavelengths. *J. Am. Chem. Soc.* **2017**, *139*, 9333-9340.
